# Dynamic causal communication channels between neocortical areas

**DOI:** 10.1016/j.neuron.2022.05.011

**Published:** 2022-08-03

**Authors:** Mitra Javadzadeh, Sonja B. Hofer

**Affiliations:** 1 Sainsbury Wellcome Centre for Neural Circuits and Behaviour, University College London, London, UK

**Keywords:** visual cortex, inter-areal communication, neocortical interactions, visual processing, population activity dynamics, causal manipulation, communication subspace

## Abstract

Processing of sensory information depends on the interactions between hierarchically connected neocortical regions, but it remains unclear how the activity in one area causally influences the activity dynamics in another and how rapidly such interactions change with time. Here, we show that the communication between the primary visual cortex (V1) and high-order visual area LM is context-dependent and surprisingly dynamic over time. By momentarily silencing one area while recording activity in the other, we find that both areas reliably affected changing subpopulations of target neurons within one hundred milliseconds while mice observed a visual stimulus. The influence of LM feedback on V1 responses became even more dynamic when the visual stimuli predicted a reward, causing fast changes in the geometry of V1 population activity and affecting stimulus coding in a context-dependent manner. Therefore, the functional interactions between cortical areas are not static but unfold through rapidly shifting communication subspaces whose dynamics depend on context when processing sensory information.

## Highlights

• Optogenetic perturbations reveal the causal structure of long-range cortical influences
• How visual areas influence each other changes dynamically over tens of milliseconds
• Feedback to V1 improves visual stimulus encoding required for behavior
• The dynamics of feedback influences depend on the behavioral context

## Teaser

How do brain regions influence each other? Javadzadeh and Hofer use a causal approach to study cortical interactions. They find that communication subspaces between areas—the channels by which they interact—are not static over time but re-organize rapidly over timescales as short as tens of milliseconds.

## Introduction

Animal behavior arises from distributed patterns of neural activity across many brain regions. Neural activity in any one region does not develop in isolation but is constantly shaped by, and in turn shapes, the activity in others. Hence, brain-wide activity patterns underlying behavior evolve on a moment-to-moment basis through the continuous exchange of information between brain areas. Understanding such complex networks of information flow requires looking beyond the anatomical connections that provide the substrate for communication and asking how distributed brain areas dynamically influence each other (Battaglia et al., 2012; Friston, 2011; Park and Friston, 2013).

Statistical relations of activity across brain regions have been traditionally used to infer causal interactions between them (Semedo et al., 2020; Kang and Druckmann, 2020; Keeley et al., 2020; Aertsen et al., 1989; Siegel et al., 2012; Singer and Gray, 1995; Gerstein and Perkel, 1969; Womelsdorf et al., 2007; Fries, 2015). The majority of previous studies have relied on macroscopic measures of neural activity, such as the local field potential, EEG, or fMRI BOLD signals (Rogers et al., 2007; Srinivasan et al., 2007; Hutchison et al., 2013; Gonzalez-Castillo and Bandettini, 2018; Gregoriou et al., 2009; Bosman et al., 2012; Bastos et al., 2015). Such one-dimensional summary signals reflect combined activity from many neurons. However, several recent studies have emphasized that specific patterns of population activity, rather than the average level of activity in an area, are crucial for how it relates to the activity of downstream targets (Kaufman et al., 2014; Kohn et al., 2020; Semedo et al., 2019). For instance, in the motor cortex of macaques, only certain activity patterns are predictive of muscle movement, whereas other patterns fall into a “null space” and are not related to movement (Kaufman et al., 2014). Likewise, in the macaque visual cortex, only a small subset of population activity patterns is statistically related to downstream activity (Semedo et al., 2019). Such findings have highlighted the importance of single-cell resolution measurements in characterizing inter-areal interactions and have spurred the development of diverse multivariate statistical analysis methods to quantify the interactions between populations of neurons recorded across different brain areas (Kang and Druckmann, 2020; Keeley et al., 2020; Semedo et al., 2020).

However, despite the recent advances in multi-area population recording and analysis methods, the main challenge in quantifying inter-areal communication remains unresolved: the correlated patterns of activity measured across two brain areas could arise from the causal influences that these areas have on each other or, alternatively, could be due to common or correlated inputs to both areas and, therefore, not be reflective of causal interactions. It has been shown that statistical approaches cannot distinguish these possibilities (Semedo et al., 2020; Reid et al., 2019; Das and Fiete, 2020; Mehler and Kording, 2020). This problem is particularly pronounced in highly interconnected networks with a large number of unobserved sources of variability, such as other brain regions that were not recorded (Das and Fiete, 2020). Therefore, although previous studies have shown that the statistical relations of simultaneously recorded neural activity in neocortical areas are modified depending on behavioral demands (Park and Friston, 2013; Hutchison et al., 2013; Gonzalez-Castillo and Bandettini, 2018; Gregoriou et al., 2009; Bosman et al., 2012; Ruff and Cohen, 2016; Buschman and Miller, 2007), we do not yet know whether this is reflective of changes in causal influences between areas.

Overcoming this long-acknowledged obstacle in the field requires a causal approach to measuring inter-areal interactions, based on manipulations of neural activity. Local cooling to inactivate a specific brain area has been used to assess the influence of cortical areas on each other (Hupé et al., 1998; Nassi et al., 2013; Gómez-Laberge et al., 2016). However, although such approaches capture causal interactions, they do not have the fine temporal resolution necessary for capturing instantaneous influences and their dynamics. More recently, optogenetic methods have enabled temporally precise manipulation of neural activity in order to examine the effect of silencing or activating different brain areas on long-range targets (for instance, Kim et al., 2017; Chabrol et al., 2019; Finkelstein et al., 2021; Goldbach et al., 2021; Jin and Glickfeld, 2020; Sauerbrei et al., 2020). However, studies using this approach have not focused on how communication between populations of neurons is dynamically organized or how it causally shapes neural activity patterns at different time points or in different behavioral contexts.

Accordingly, the principles of how distributed neuronal populations communicate are still unclear. Specifically, it remains unresolved how the patterns of influence from one area on its target populations in other areas are structured, whether these causal influences are static or change over time, and whether communication channels between areas can be dynamically regulated depending on the behavioral state of the animal.

We addressed these questions, focusing on the interactions between primary visual cortex (V1) and lateromedial area (LM) in mice. Area LM is considered the homolog of secondary visual cortex (V2) in primates (Wang and Burkhalter, 2007). It is the largest higher visual area in the mouse cortex and most densely innervated by V1 (Glickfeld and Olsen, 2017). LM neurons have, on average, slightly larger receptive fields than V1 neurons; otherwise, visual response properties in the V1 and LM are broadly similar (Glickfeld and Olsen, 2017; Wang and Burkhalter, 2007), and both V1 and LM have been shown to be important for visual discrimination (Poort et al., 2015; Resulaj et al., 2018; Goldbach et al., 2021; Jin and Glickfeld, 2020). Investigating causal interactions between V1 and LM, therefore, provides a suitable model for studying feedforward and feedback communication between different levels of the visual hierarchy.

## Results

### Measuring causal interactions between neocortical areas

**Figure 1:**
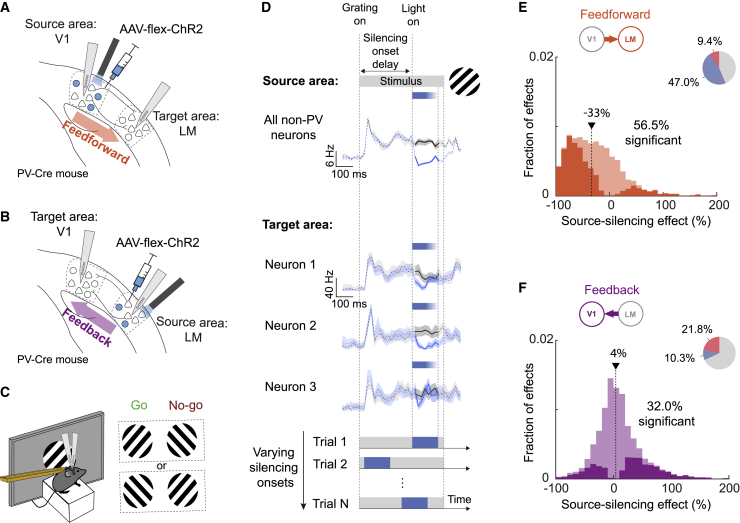
Experimental design for measuring causal influences between V1 and LM (A) Paired recordings in retinotopically matched regions of V1 and LM with silicon probes in PV-Cre mice. In order to measure the influence of V1 on LM cells (feedforward influence), V1 was silenced through light-mediated activation of parvalbumin-expressing inhibitory cells expressing ChR2 after injection of AAV-flex-ChR2. (B) As in (A) but with optogenetic silencing of LM in order to assess influence of LM on V1 (feedback influence). (C) Recordings and optogenetic manipulation were performed during a go/no-go task. Head-fixed, stationary mice were presented with two differently oriented stationary grating stimuli (45° or −45°), only one of which was rewarded. The identity of the rewarded stimulus was randomized across mice. Mice reported the rewarded stimulus by licking a spout, which triggered the delivery of the reward. (D) Example traces of spiking activity during V1 silencing. The traces denote stimulus-evoked activity in the source (V1) and target (LM) area in control (black traces) and silencing trials (blue traces), binned at 20 ms. Shading is the 95% bootstrapped confidence interval. The gray bar on top indicates the presence of the visual grating stimulus (45° or −45° orientation, 40° diameter). Blue bars indicate the time of optogenetic silencing. Top, average activity of all non-PV neurons (broad action potential waveform) in the source area (V1). Middle, average activity in control and silencing trials of three example neurons in the target area (LM). Bottom, dynamic characterization of inter-areal influence by varying silencing onset times. For each trial, the silencing onset time is chosen in a randomized manner from a pool of 8 values, tiling the stimulus duration with ∼65-ms resolution. (E) Distribution of the effects of silencing V1 on the firing rate of LM neurons from all silencing time windows and stimuli (feedforward influences, n = 278 neurons, 7 mice, 8 silencing time windows) calculated as a percentage change in firing rate when silencing V1. Negative source-silencing effects indicate decreases in firing rate during silencing and, therefore, a net excitatory influence of V1 on the LM cell. Positive source-silencing effects indicate increases in firing rate and, therefore, a net inhibitory influence of V1 on the LM cell. Significant effects are shown in bright colors (56.5% of all effects). The arrow denotes the median of the distribution. The pie chart shows the fraction of significant increases (red) and decreases (blue) in LM neuron firing rates during V1 silencing. (F) As in (E), but distribution of the effects of silencing LM on the firing rate of V1 neurons (feedback influences, n = 242 neurons, 7 mice, 8 silencing time windows, 32.0% of all effects significant). See also Figures S1–S4.

We developed a paradigm for measuring causal interactions between cortical areas by manipulating neuronal activity and characterizing inter-areal communication on the neuronal population level with single-cell resolution. Using direct experimental manipulations to establish causal interactions eliminated the need for relying on statistical associations of neural activity. We measured the directional influence of one cortical area (source) on another (target), focusing on feedforward and feedback influences between V1 and LM in mice (Figures 1A and 1B). We performed simultaneous electrophysiological multi-channel recordings at retinotopically matched locations in the two areas (Figures S1A–S1C). We then silenced one of the two areas for brief time windows using optogenetic activation of inhibitory parvalbumin-expressing interneurons expressing channelrhodopsin-2 (ChR2) (Cardin et al., 2009; Guo et al., 2014) and measured the instantaneous effect on the recorded neurons in the target area, revealing the causal influence of the source area on the target neurons’ activity.

In order to assess how behavioral state modulates cortical communication, we trained mice to perform a go/no-go task. Animals learned to discriminate two stationary visual grating stimuli of which only one was rewarded (Figures 1C, S1D, and S1E). Optogenetic silencing was performed in 150-ms time windows during the presentation of the 500-ms-long grating stimuli. The onset of silencing varied in each trial, tiling the duration of the visual stimulus in ∼65-ms steps (Figure 1D). The effect of silencing on target area firing rates was only assessed during the time window of optogenetic manipulation. This approach allowed us to quantify how the influence of the source on the target area may change over time during the cortical processing of behaviorally relevant stimuli with a resolution of tens of milliseconds (Figure 1D).

### Functional organization of inter-areal influences

We first characterized the average influence of V1 activity on LM neurons (feedforward influence), irrespective of silencing time window and task condition. We measured the trial-averaged percentage of change in the firing rate of LM neurons during each time window in which V1 was silenced. A large fraction of LM neurons was suppressed by V1 silencing (Figure 1E; median change in spiking rate = −33%, with 54.9% ± 7.6%, mean ± SEM of neurons significantly affected in at least one time window), confirming a predominantly excitatory feedforward influence of V1 on this higher visual area. The extent to which LM neurons were suppressed when V1 was silenced was partly related to their average firing rate, the cortical depth at which they were located, and their relative receptive field position, according to a multivariate regression model (Figure S2; see STAR Methods). Specifically, LM neurons were less affected by V1 activity when they resided in deep cortical layers (Yang et al., 2013) or when their spatial receptive field was far displaced from the retinotopic location of the center of optogenetic manipulation in V1 (Figures S2D and S2E). We also examined how the influence of V1 on LM cells depended on their responses during the discrimination task. The influence of V1 silencing was independent of the visual stimulus preference (preferential response to the go or the no-go stimulus) or behavioral choice preference (preferentially active during lick response or absence of lick response) of LM neurons (Figures S3A and S3D). However, we found that V1 silencing caused stronger suppression in more visually selective LM neurons (neurons with a higher difference in response to the go and no-go stimulus; see STAR Methods), indicating that V1 is driving selective responses to the task-relevant stimuli in LM (Figures S3A and S3C). These results indicate that feedforward input from V1 exerts a structured influence on LM, depending on the neurons’ retinotopic position and visual response tuning.

We then performed equivalent experiments while silencing higher visual area LM to measure the effect of LM feedback on the activity of V1 neurons. Silencing LM changed the firing of a substantial number of V1 neurons (Figure 1F; 46.9% ± 6.9%, mean ± SEM of neurons significantly affected in at least one time window) but did not have a clear net excitatory or inhibitory influence (median change in spiking rate of 4%; Figure 1F). Instead, LM activity had diverse effects on individual V1 neurons, including decreased or increased activity upon LM silencing. A previous study in awake primates found similarly diverse excitatory and inhibitory influences of higher visual area V2 on V1 (Nassi et al., 2013). In contrast to the feedforward effect of V1 on LM, we could not identify any anatomical, physiological, or visual response attributes of V1 neurons that could explain by how much or in which direction they were affected by the feedback from LM (Figures S2C, S2F, S2G, S3B, S3E, and S3F).

### Effect of silencing on stimulus decoding and behavior

Next, we examined how feedforward and feedback interactions between V1 and LM affect the cortical representations of the two visual stimuli mice had to discriminate to perform the task. Source-area silencing had, on average, a similar effect on the response to the rewarded grating stimulus (during go trials) and the non-rewarded stimulus (during no-go trials), both in LM during the silencing of feedforward input from V1 and in V1 during the silencing of feedback from LM (Figure S4, go versus no-go feedforward influence p = 0.87, feedback influence p = 0.78, two-sided Wilcoxon rank-sum test).

**Figure 2:**
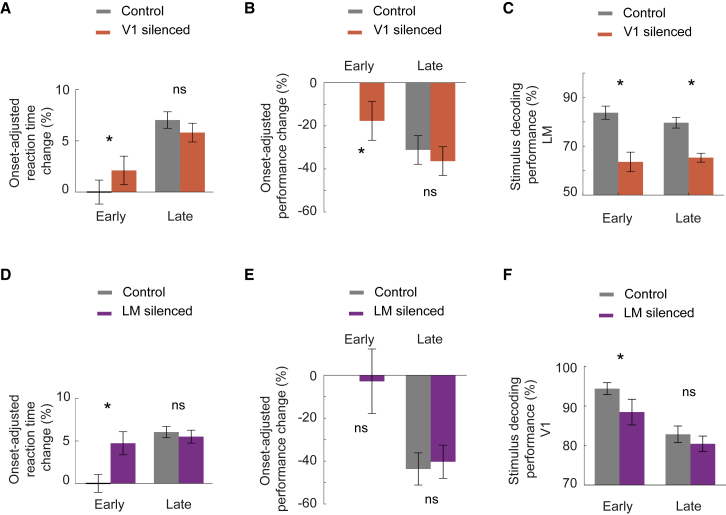
The role of V1-LM interactions in visual discrimination (A) Percentage of change in the onset-adjusted reaction time, compared with the average reaction time of each mouse (see STAR Methods), calculated separately in control (gray) and V1 silencing (orange) trials with either early (<100 ms) or late (>100 ms) silencing onset. p = 0.049 for early silencing and p = 0.215 for late silencing, using two-sided Wilcoxon rank-sum test. (B) Percentage of change in the onset-adjusted behavioral d-prime, compared to the average d-prime (see STAR Methods), calculated separately in control (gray) and V1 silencing (orange) trials with either early (<100 ms) or late (>100 ms) silencing onset. p = 0.035 for early silencing and p = 0.123 for late silencing, from Wilcoxon two-sided signed-rank test. (C) Decoding performance of a linear classifier distinguishing go from no-go trials (including only correct trials) using LM population activity (see STAR Methods). The classifier performance was measured on test control trials (gray) and V1 silencing trials (orange) and calculated separately for early silencing time windows with silencing onsets before the beginning of the behavioral response window (<100 ms after stimulus onset, early) and later silencing time windows (>100 ms, late). p < 10^−3^ for early silencing and p < 10^−4^ for late silencing, using Wilcoxon two-sided signed-rank test. (D) As in (A), but with and without silencing LM. Difference in reaction time: p = 0.036 for early silencing and p = 0.108 for late silencing, using two-sided Wilcoxon rank-sum test. (E) As in (B), but with and without silencing LM. Difference in d-prime: p > 0.05 for both early and late silencing, using Wilcoxon two-sided signed-rank test. (F) As in (C), but using the activity of V1 neurons, with and without LM silencing. p = 0.007 for early silencing and p = 0.11 for late silencing, using Wilcoxon two-sided signed-rank test. Error bars depict the standard error of the mean (SEM) in all panels. See also Figure S5.

But how is neural decoding of the two stimuli affected in the two cortical areas by silencing feedforward or feedback input? And how does this impact the animals’ discrimination performance? Consistent with previous studies (Goldbach et al., 2021; Jin and Glickfeld, 2020; Resulaj et al., 2018), the silencing of either V1 or LM early after stimulus onset (silencing onset < 100 ms after stimulus onset, before behavioral responses to the go stimulus occurred) degraded animals’ performance in the discrimination task (Figures 2A, 2B, 2D, 2E, and S5). To quantify neural discrimination performance, we used a linear decoder to classify whether a go or a no-go trial had occurred from the population activity in either V1 or LM (see STAR Methods). Silencing V1 significantly reduced stimulus decoding accuracy in LM throughout the visual stimulus presentation (Figure 2C; p values < 10^−3^), likely due to the strong suppression of average responses in LM during V1 silencing (Figure S5C; p < 0.02, two-sided Wilcoxon signed-rank test). More surprisingly, even though silencing LM did not change the average level of activity in V1 (Figure S5F), it significantly reduced the accuracy of decoding visual stimulus identity from V1 population activity. However, silencing LM only degraded stimulus decoding accuracy during the early stimulus period, before behavioral responses were apparent (Figure 2F, early: p = 0.007; late: p = 0.11, Wilcoxon two-sided signed-rank test). Interestingly, this is the stimulus period during which V1 activity is crucial for the animals’ behavioral decision (Figures 2A and 2B; Resulaj et al., 2018).

### Inter-areal influences vary over time

**Figure 3:**
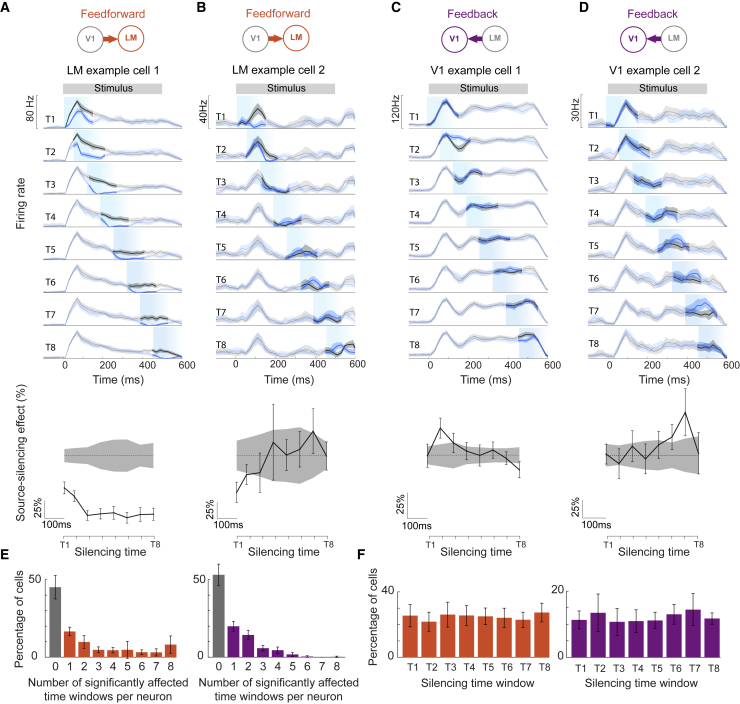
Temporal profile of feedforward and feedback influences on example target neurons in V1 and LM (A and B) Top, firing rates of two example LM neurons in control (black) and V1 silencing (blue) trials during go stimuli at 8 different silencing onset times (T1–T8). The gray bar on top indicates the presence of the visual grating stimulus. Light blue shading marks the timing and duration of optogenetic silencing. PSTHs were smoothed over 60 ms using a moving average filter. Shading indicates the 95% bootstrapped confidence intervals of the mean. Bottom, feedforward source-silencing effects on the target neuron over time calculated as a percentage change in firing rate when silencing the source area. Zero is indicated by a dashed line. Chance levels are indicated by gray shading and calculated as 95% confidence interval of percentage differences in the firing rate of bootstrapped control trials. Error bars indicate 95% bootstrapped confidence interval of the mean. (C and D) As in (A and B) but showing effects of silencing LM on two example V1 cells. (E) Distribution of the number of time windows in which individual neurons were significantly affected (after Bonferroni correction for 8 comparisons) by V1 silencing (left, LM neurons, n = 278 neurons, 7 mice) and by LM silencing (right, V1 neurons, n = 242 neurons, 7 mice). Data was averaged across go and no-go trials. Error bars depict 95% confidence interval (2 × SEM) of the mean across animals. (F) Percentage of neurons showing a significant change of firing rate during each silencing time window for LM neurons during V1 silencing (left, one-way ANOVA, p = 0.9) and for V1 neurons during LM silencing (right, one-way ANOVA, p = 0.8). Error bars as in (E).

Quantification of average source-silencing effects (Figures 1E and 1F) on the target area does not reveal how individual neurons are influenced at different moments in time. To determine how the two cortical areas influenced each other over the course of visual stimulus presentation, we compared how single-cell activity in the target area was affected in eight different silencing time windows (T1–T8) tiling the visual stimulus duration (Figures 1D and 3). In some target area neurons, the change in firing rate induced by source-area silencing was relatively similar during all time windows (Figure 3A), revealing a constant influence of the source area on these neurons over time. By contrast, many neurons showed varying changes in their firing rates during different silencing windows (Figures 3B–3E). For instance, the feedback from LM often exerted a significant effect on a V1 neuron only during a short time period (as short as <100 ms; Figures 3C–3E). These silencing effects were consistent from trial to trial for the same neuron, but not consistent between neurons, with individual cells affected at different times during the stimulus presentation (Figure 3F).

**Figure 4:**
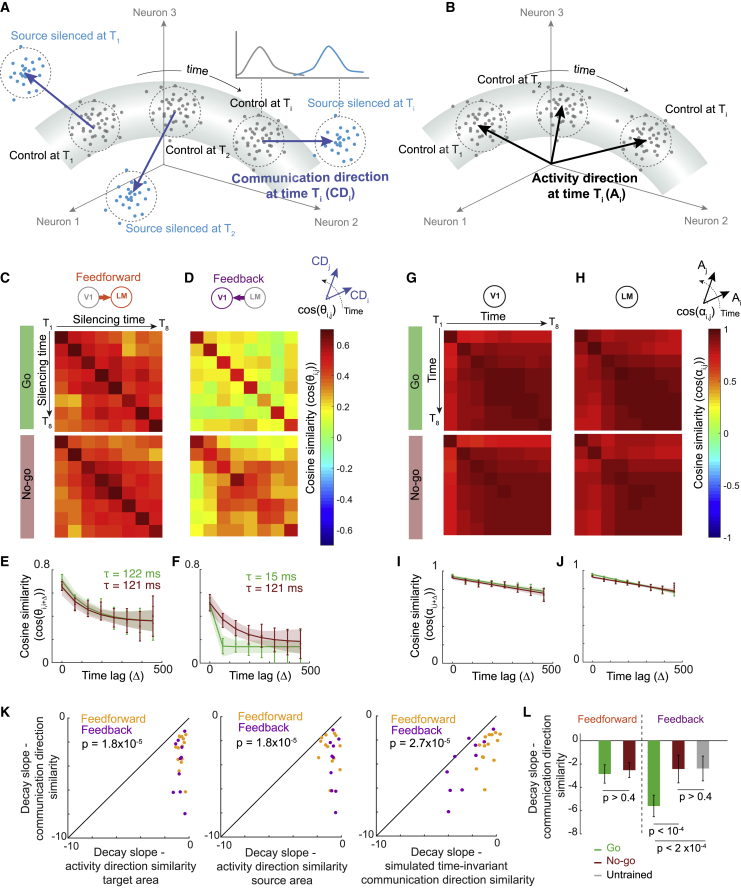
Population-level dynamics of inter-areal influences (A) Schematic of the population analysis framework. Each point in the neural activity space specifies the population activity in the target area in a given time window (150 ms) of one trial. Gray points denote control trials and blue points source-silencing trials. The communication direction (blue arrows) is calculated for each time window as the direction that maximally separated the activity in control trials (gray cloud of points) from that in source-silencing trials (blue cloud of points; see STAR Methods). (B) Activity directions (black arrows) are calculated for each time window as the direction specifying the center of the trial-averaged activity in control trials in that time window (center of the gray cloud of points). (C) Matrices depicting cross-validated cosine similarity between the communication directions, as shown in (A), of all pairs of silencing time windows during the go (top) and no-go stimulus (bottom) for feedforward influences on LM (silencing of V1). Matrices are averaged across animals (n = 7 mice). (D) As in (C), but for the feedback influences on V1 (silencing of LM) (n = 6 mice). (E) Cosine similarity of pairs of feedforward communication directions in different time windows as a function of the time lag between them during the go (green) and no-go (red) stimuli. Error bars depict the 95% confidence interval of the mean (2× SEM). Lines depict exponential fits, and shading shows 95% prediction bounds of the fits. The decay time constants of the exponential fits (τ) are shown for go (green) and no-go (red) stimuli. (F) As in (E) but for feedback communication directions. (G) Matrices depicting cross-validated cosine similarity between activity directions (as shown in B) in V1 (n = 13 mice) of all pairs of time windows in control trials during the go (top) and no-go (bottom) stimuli. (H) As in (G) but showing the cross-validated cosine similarity of activity directions in LM (n = 13 mice). (I) Pairwise cosine similarity of V1 activity directions in different time windows as a function of the time lag between them during the go (green) and no-go (red) stimuli. Error bars depict the 95% confidence interval of the mean (2×SEM). Lines depict linear fits, and shading shows 95% prediction bounds of the fits. (J) Same as (I), but for LM activity directions. (K) Relationship between the initial slopes (slopes between lag 0 and lag 1) of the decay over time lags of communication direction similarities and activity direction similarities in the target area (left) and source area (middle) and between initial decay slopes of communication direction similarities in the real dataset and the simulated dataset assuming time-invariant inter-areal influences (right). p values from two-sided Wilcoxon signed-rank tests. Orange and purple dots show data from individual animals during V1 silencing (feedforward influences) and LM silencing (feedback influences) experiments, respectively. (L) Initial slopes (slopes between lag 0 and lag 1) of the decay over time lags of feedforward and feedback communication directions in go (green) and no-go (red) trials of trained animals and of feedback communication directions in untrained animals (gray). Error bars depict the 95% confidence interval of the mean (2 × SEM). p values from Wilcoxon two-sided signed-rank test for comparisons between go and no-go in trained animals and from two-sided Wilcoxon rank-sum test for comparisons with untrained animals. See also Figures S6–S8.

To quantify the diverse and temporally variable contributions of a source area to the target neurons’ activity, we derived a population-level measure of inter-areal influence between V1 and LM. In a given time window, the population activity of *n* simultaneously recorded neurons in the target area can be described as a point in an *n*-dimensional space in which the coordinates specify the firing rate of each neuron. In this framework, one set of points represents the population activity during a specific time window in all control trials without optogenetic manipulation, and another set of points constitutes the population activity in all silencing trials in which source-area activity was absent during the same time window (Figures 4A and 4B). To find the relation between these population activity states, we identified the direction in the *n*-dimensional activity space along which the population activity during source-silencing trials could be maximally discriminated from control trials using linear discriminant analysis (LDA) (Fisher, 1936) (see STAR Methods; Figures S6A–S6F). This direction denotes a mode of target area activity (pattern of activity in a specific neural ensemble), which is causally dependent on the activity in the source area and is, therefore, termed the causal communication direction (Figure 4A).

We determined causal communication directions separately for the eight time windows during the visual stimulus and quantified their pairwise similarity by calculating the cosine similarity between communication directions (the dot product of unit-length vectors) for each pair of silencing time windows. Cosine similarities were calculated in a cross-validated manner (see STAR Methods) and, therefore, provided a robust estimate of similarity with respect to trial-to-trial variabilities. We visualized cross-validated cosine similarities between all possible pairs of communication directions of all silencing time windows T1 to T8 in cosine similarity matrices (Figures 4C and 4D) and quantified how fast communication directions changed by comparing the similarity of directions over all time lags (Figures 4C–4F). This analysis allowed us to address how stable the causal communication direction was over time and thereby assess the time-varying causal influence that neuronal populations in the two cortical areas had on each other.

The similarity of both feedforward and feedback communication directions decayed relatively rapidly with time (Figures 4E and 4F, exponential decay time constant (τ) < 122 ms for all conditions), indicating that both feedforward and feedback influences on population activity in V1 and LM are temporally dynamic. To test whether this time-varying influence can simply be explained by temporally variable population activity in the source or target area, we compared how the patterns of population activity within V1 and LM changed over time. The trial-averaged population activity at each time window was quantified using population activity directions, defined as the center coordinate of the set of points in the population activity space corresponding to activity in control trials at that time window (Figure 4B). We calculated cross-validated cosine similarities between the activity directions of control trials of all eight time windows (Figures 4G–4J). The modes of activity in both V1 and LM were very similar across different time windows, and the similarity of activity directions, therefore, decayed much more slowly over time than the similarity of both the feedforward and feedback communication directions (Figure 4K, left and middle, all p values < 2 $\times 10^{-5}$, two-sided Wilcoxon signed-rank test; see also Figure S8F for the same analysis with baseline-subtracted visual responses). This was the case even if only the 20% of source-area neurons with the most time-varying activity were included for calculating activity directions (Figures S6G–S6N; see STAR Methods). Therefore, the fast changes in communication directions cannot be explained by a subset of source-area neurons with particularly high temporal variations in their firing rate providing input to the target area.

However, the above analyses rely on comparisons between LDA vectors capturing manipulation effects and population activity vectors, which are different measures with different magnitudes and different levels of robustness to trial-to-trial noise (cross-validated similarity at time lag 0; see STAR Methods). To corroborate that none of these differences can explain the faster decay in the similarity of communication directions compared with activity directions, and to enable a more direct comparison between the changes in activity and communication directions over time, we generated an artificial dataset to simulate how similar communication directions of different time windows would be if source-silencing effects on individual neurons were invariant in time. For each target neuron, we used its experimentally measured activity in control trials and simulated the activity in source-silencing trials, such that the effect of silencing was the same in each time window while maintaining the experimentally observed distribution of silencing effects per cell (see STAR Methods). This allowed us to directly compare changes in communication directions with and without time-varying influences of silencing, while preserving measured neuronal activity dynamics and the diversity of single-cell silencing effects, as well as experimentally observed trial-to-trial variability of firing rates. The communication directions of this simulated dataset changed slower over time than those of the experimental data (Figure 4K, right, p = 2.7 × 10^−5^, two-sided Wilcoxon signed-rank test; Figures S7A and S7B), confirming that the fast decay in the similarity of communication directions cannot be explained by trial-to-trial noise or the temporal dynamics of neuronal activity patterns.

In addition to the communication directions, which describe the patterns of influence on the target neurons, we also quantified the magnitude of the influence on the target area’s population activity for both feedforward and feedback communication. We measured the high-dimensional distance between target area population activity in control and source-silencing trials (see STAR Methods) and found that, interestingly, these distances do not change significantly over time (Figures S7C and S7D, all p values > 0.4, one-way ANOVA).

Therefore, our data show that communication between cortical areas is dynamic and reorganizes over time. Although the magnitude of how strongly one area influences population activity in another area stays constant, different patterns of influence are exerted by the source area at different time points during the processing of a visual stimulus.

### Temporal dynamics of feedback influences depend on behavioral relevance

Previous studies have shown that the relationship between neuronal activity in different cortical areas during processing of a sensory stimulus is modulated by the relevance and behavioral consequences of the stimulus (Bosman et al., 2012; Ferezou et al., 2007; Gregoriou et al., 2009; Pinto et al., 2019; Ruff and Cohen, 2016). These results raise the possibility that information flow between areas can be flexibly adjusted by the behavioral context of sensory signals. To determine whether the causal influence of areas V1 and LM on each other changes depending on behavioral task demands, we compared how the time-varying effects of feedforward and feedback input on population activity differed in go and no-go trials during the visual discrimination task. Only the go stimulus was associated with a reward and was, therefore, behaviorally most relevant.

The temporal dynamics of feedforward communication directions, denoting how much the activity mode in LM that was causally dependent on activity in V1 changed over time, were similar during the go and no-go stimulus (Figures 4E and 4L, exponential decay time constant, go trials: 122 ms, 81–153 ms, 95% confidence interval [CI]; no-go trials: 121 ms, 89–170 ms CI; p = 0.99, permutation test). In contrast, the dynamics of feedback influences were much faster during the go than the no-go stimulus, with the cosine similarity of feedback communication directions from LM to V1 decaying at a time constant of ∼15 ms only during the go stimulus (Figures 4F and 4L, exponential decay time constant, go trials: 15 ms, 0–46 ms CI; no-go trials: 121 ms, 93–163 ms CI; p = $1.3\;\times \;10^{-4}$, permutation test). These effects could not be explained by differences in V1 or LM activity, since the dynamics of population activity directions in both areas were similar during go and no-go trials (Figures 4G–4J and S7E), as was the level of trial-to-trial variability of communication directions (Figure 4F, time lag 0; see STAR Methods; go versus no-go trials, p > 0.5, two-sided Wilcoxon signed-rank test). Moreover, feedback communication directions changed faster during go trials, irrespective of the identity of the go stimulus (Figure S7H), showing that the observed differences were due to the contingency of the stimuli.

To rule out that differences in feedback communication could be due to differences in motor actions during go and no-go trials such as licking, we compared the dynamics of feedback influences during an early epoch before mice responded to the visual stimulus (50–200 ms post stimulus onset) and a late epoch in which mice showed lick responses to the go stimulus (>250 ms post stimulus onset). In both epochs, changes in feedback communication directions were faster during go stimulus trials (Figure S7F).

These data indicate that feedback communication during sensory processing is regulated by behavioral relevance. Accordingly, we found that in untrained mice that were passively viewing the visual stimuli, feedback communication directions exhibited temporal dynamics similar to those during no-go trials in trained animals (Figures 4L and S7G). Hence, the ensembles of V1 neurons influenced by LM activity changed much more rapidly when the visual stimulus processed by these cortical areas is predictive of reward (see also Figure S8A). Although we did not find similar effects of stimulus relevance on the temporal patterns of feedforward communication, the influence of V1 onto downstream targets may be modulated by other behavioral variables not captured by our task, such as spatial attention (Bosman et al., 2012; Gregoriou et al., 2009; Ruff and Cohen, 2016).

### Feedback restructures V1 covariance patterns depending on behavioral relevance

How does the dynamic influence of feedback impact activity patterns in V1? Since trial-averaged V1 activity directions changed at similar rates during the stimulus in go and no-go trials (Figures 4I, 4J, and S7E), we looked beyond average activity patterns and examined the geometry of trial-to-trial fluctuations. Neurons in cortical areas form functional subnetworks within which activity is correlated from trial to trial. The organization of these co-fluctuations—the covariance structure of population activity—has important implications for the coding capacity and readout of information from an area (Moreno-Bote et al., 2014; Rosenbaum et al., 2017; Shamir and Sompolinsky, 2004). These co-fluctuations can be captured by principal component analysis, wherein the first few principal components (PCs) define the most prominent modes of correlated activity in V1. We computed the first three PCs of V1 population activity (explained variance = 72% ± 9.2%, mean ± SD) separately for the eight time windows during visual stimulus presentation and used cross-validated cosine similarity to compare their differences over time, as described above (see STAR Methods).

**Figure 5:**
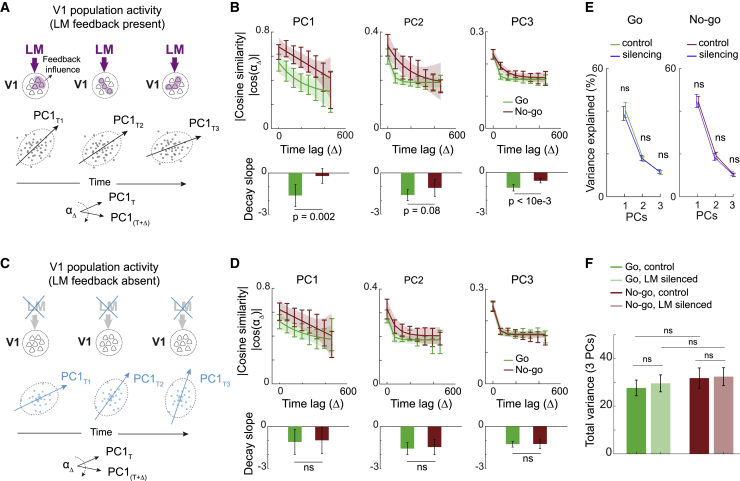
Feedback modulates the covariance structure of V1 population activity (A) Schematic of V1 population activity in different time windows in control trials. The cloud of points indicates the trial-to-trial variability in the patterns of V1 population activity. The shape of this cloud is described by the principal components. Principal component (PC) directions were calculated separately for each time window, and their similarity was compared over time. (B) Top, absolute value of pairwise cross-validated cosine similarity of the first (left), second (middle), and third (right) PCs of V1 activity in different time windows as a function of the time lag between them in go (green) and no-go (red) trials. Error bars depict the 95% confidence interval of the mean (2×SEM). Lines depict exponential fits, and shading shows 95% prediction bounds of the fits. Bottom, initial slope (slope between lag 0 and 1) of the decay of principal component similarity over time lags for go and no-go trials. Error bars depict the 95% confidence interval of the mean (2×SEM). p values from two-sided Wilcoxon signed-rank tests. (C) As in (A), but during silencing of LM, depicting PCs of V1 population activity over time in the absence of LM feedback. (D) As in (B), but during LM silencing trials (in the absence of LM feedback to V1). The difference between the decay slopes of PC similarity in go versus no-go trials was significantly reduced compared with the control trials shown in (B) (p = 0.03, Wilcoxon signed-rank test, pooled across the first 3 PCs). (E) Percentage of variance explained by each of the first three PCs in V1 during go (green, left) and no-go (red, right) stimuli in control and silencing trials (blue) across all time windows. Error bars depict the 95% confidence interval of the mean (2×SEM). p values from two-sided Wilcoxon signed-rank tests. (F) The sum of variance across the first three PCs in V1 for control and LM silencing trials, during the go (green) and no-go (red) stimuli, averaged across all time windows. Error bars depict the 95% confidence interval of the mean (2 ×SEM). p values from two-sided Wilcoxon signed-rank tests. See also Figure S9.

Interestingly, covariance patterns of V1 activity were not stable but appeared to re-organize over time, as the dominant PCs changed their direction in succeeding time windows (Figures 5A and 5B). Moreover, the PCs changed direction at a faster rate in go compared with no-go trials, revealing that functional V1 subnetworks re-organized faster during the presentation of the behaviorally relevant go stimulus. However, this was only the case in control trials when LM feedback was intact (Figures 5A and 5B). During silencing trials, when the feedback from LM was absent, this difference was significantly reduced (p = 0.03, Wilcoxon signed-rank test, pooled across the first 3 PCs), and the temporal evolution of PCs was similar in go and no-go trials (Figures 5C and 5D), indicating that the temporal evolution of V1 covariance patterns depended on the feedback from higher visual area LM. In contrast, how much of the variance of V1 activity each PC could explain and their total variance was not different in go and no-go trials and was not changed when LM was silenced (Figures 5E and 5F; see STAR Methods), indicating that feedback only rotated the covariance structure, without affecting the overall amount of trial-to-trial variability in V1 population activity.

Given that feedback regulates the correlation structure of V1 population activity, we asked how these influences were reflected in the temporal and pairwise dependencies of firing rates at the single neuron level. Temporal dependencies in the firing rate of individual V1 neurons over time, quantified by the autocorrelation function over different time lags, were also different during go and no-go stimuli, as autocorrelations were lower during go stimuli (Figure S9A). This difference was absent when the feedback from LM was silenced (Figure S9A). Moreover, V1 neurons whose firing rate was more strongly affected by LM silencing showed faster decay of autocorrelations (Figure S9B), confirming that the decreased dependency of neuronal firing rates with time was related to the feedback from LM. Finally, pairwise noise correlations between V1 neurons were on average weaker in go than in no-go trials (Figure S9C), confirming previous findings of decreased firing rate interdependencies in cortical areas during the processing of behaviorally relevant visual stimuli (Cohen and Maunsell, 2009). However, the feedback from LM did not affect the average strength of pairwise noise correlations nor their modulation by task demands (Figures S9C and S9D).

Therefore, the feedback from LM affects specific aspects of V1 population activity patterns. Feedback renders correlation patterns in neuronal firing rates more dynamic over time without affecting the overall variability of population activity and, therefore, contributes to the continual restructuring of functional V1 subnetworks during stimulus coding. Moreover, the rate at which V1 functional subnetworks re-organize is modulated by behavioral demands through a change in the temporal dynamics of the feedback influences from LM.

## Discussion

By using a causal approach to study inter-areal communication in the neocortex, we quantified the dynamics of feedforward and feedback interactions between cortical visual areas. We found that the patterns of influence on the target population can change rapidly, with different subsets of neurons affected at different moments in time within tens of milliseconds. This was in stark contrast to the stable activity patterns elicited by visual stimulation within the areas themselves. Moreover, the dynamics of inter-areal communication were modulated by the animal’s behavioral state.

### Functions of dynamic communication

We found that cortical areas exert influences on each other along communication dimensions that continually change direction, although the magnitude of these influences is constant over time (Figures 4, S7C, and S7D). Our results imply that these dynamics in inter-areal communication serve an important function since their speed rather than the average strength of influence is regulated by behavioral demand (Figures 4D–4F and S4). What potential function can these dynamics serve, and what advantages do they offer for cortical computation?

Specific patterns of population activity have been shown to be important for encoding various task-relevant variables (Kaufman et al., 2014) such that the activity along these “coding dimensions” would lead to behavioral effects, while other patterns of activity along the “null dimensions” would be inconsequential for the behavioral outcome. The linear decoder for classifying go and no-go trials from V1 activity (Figure 2C) corresponds to a dimension in the V1 activity space (a pattern of V1 activity) that encodes information relevant for visual discrimination. We show that silencing LM specifically affects the visual stimulus coding in V1 and animals’ behavioral performance only during the early stimulus presentation period and not later on. Importantly, the absolute magnitude of the LM feedback influence on V1 does not vary significantly over time (Figures S7C and S7D). This indicates that early during visual processing, LM influences V1 activity along the dimension that is potent for visual discrimination, while, later on, the feedback communication dimension rotates out of this alignment, ceasing its influence on visual discrimination in V1. Therefore, the rotations of communication directions could serve to momentarily align patterns of influence with different coding dimensions in the target area, allowing transient time windows of control over behaviorally relevant variables.

Moreover, cortical areas continually receive information from not just one but multiple brain areas. The rotations of communication directions of multiple converging pathways onto a cortical area could lead to their momentary alignment, forming brief windows of integration for several streams of long-range inputs. Importantly, we found that while inter-areal communication directions rotate at fast time scales, the activity directions within an area change only slowly. This implies a mechanism for flexible and fast coordination of multi-area interactions, without imposing constraints on activity dynamics.

We observed faster rotation of feedback communication directions after animals had learned the visual discrimination task and, specifically, during go trials in which the visual stimulus is associated with a reward and is, therefore, behaviorally most relevant (Figures 4D–4F). This increase in the dynamics of LM feedback influences may stem from the extra requirements imposed on cortical circuits during the performance of goal-oriented motor movements. Faster rotation of feedback communication directions could provide more precise and briefer time windows of alignment with different coding or communication directions from other areas, which may be required for more complex multi-area information routing during behavior.

### Mechanisms for dynamic communication

Which mechanisms could underlie dynamic communication on the fast time scales observed in this study? Our method measures effective connectivity between areas (Park and Friston, 2013). The described effects could, therefore, be mediated by mono-synaptic intracortical projections as well as poly-synaptic pathways, for instance, through the thalamus (Blot et al., 2021; Guillory and Sherman, 2002). Although our recordings did not provide selective access to target-projecting neurons in the source area, the population activity patterns of even the most time-varying source-area neurons could not account for the fast dynamics of inter-areal influences (Figures S6G–S6N). Inter-areal communication could be regulated by changes in the synchrony of action potential timing within or between areas (Fries, 2015; Palmigiano et al., 2017; Singer, 1999; Womelsdorf et al., 2007). Neural activity oscillations have been proposed to enable such changes in synchrony (Akam and Kullmann, 2010; Buzsáki and Draguhn, 2004; Fries, 2015). However, it is unclear how the fast dynamics in feedback influences could be achieved by long time constants required for resonance or entrainment of oscillations (Bastos et al., 2015; Buzsáki and Draguhn, 2004). Alternatively, the subpopulations of target area neurons could be rendered more or less susceptible to inter-areal influences in specific moments in time by local circuit mechanisms, non-linear amplification, or additional long-range input, for instance, from higher-order thalamic nuclei such as the pulvinar (Saalmann et al., 2012).

### The role of cortical feedback

Cortical feedback projections have been suggested to influence sensory processing in various ways (Bastos et al., 2012; Briggs, 2020; Gilbert and Li, 2013; Hupé et al., 1998; Keller and Mrsic-Flogel, 2018; Keller et al., 2020; Marques et al., 2018; Nassi et al., 2013; Nurminen et al., 2018; Pafundo et al., 2016; Vangeneugden et al., 2019), but their function is still unclear. We found that the feedback from LM increases the discriminability of visual stimuli in V1 population activity, thereby enhancing the saliency of behaviorally relevant information in V1 circuits. This influence of LM on V1 stimulus decoding is confined to the early period of visual stimulus presentation, during which V1 activity is crucial for the animals’ decision-making in the discrimination task. The dynamic nature of feedback influences, therefore, allows a temporally precise alignment of LM input with task-relevant coding dimension in V1 population activity.

More generally, we found that LM feedback can alter dependencies in V1 single-cell and population activity patterns over time. Interestingly, feedback selectively modulated the time scales of autocorrelations in V1 without affecting the average strength of pairwise noise correlations. Previous studies have reported reduced noise correlations in attentive states, hypothesized to increase the coding capacity during attention and thought to be mediated by top-down input (Cohen and Maunsell, 2009; Ramalingam et al., 2013). We observed, on average, lower noise correlations during the rewarded go stimulus, consistent with its higher behavioral relevance. However, our findings do not support a role of feedback from higher visual areas in this state-dependent modulation of noise correlations in V1, since LM feedback had no effect on noise correlation strength in either the go or the no-go stimulus condition. LM feedback may instead regulate computations in V1 by controlling the time scales of visual processing. Previous studies have shown a diversity of spike rate autocorrelation time constants across different brain areas (Cavanagh et al., 2016; Murray et al., 2014). This diversity potentially reflects area-specific temporal specializations for computations that require input integration on different time scales (Cavanagh et al., 2016; Goldman et al., 2010; Murray et al., 2014). For instance, short time scales could be beneficial for rapid detection of stimuli, while longer time scales may support computations that depend on longer integration of signals. Our findings show that these time constants can be dynamically modulated by feedback to optimize V1 circuits according to the task demands.

At the population level, we found that feedback modified the geometry of the V1 covariance structure in a context-dependent manner (Bondy et al., 2018) by rotating the PCs of V1 activity without changing the variance along each PC. The covariance structure of neural activity in a network describes the patterns of correlated variability between all recorded neurons and is, therefore, indicative of the functional subnetworks within an area. The structure of these subnetworks plays an important role in shaping the neural code and the readout of information from an area (Moreno-Bote et al., 2014; Rosenbaum et al., 2017; Shamir and Sompolinsky, 2004). Because the covariance structure relates to the connectivity between neurons (Ko et al., 2011; Rosenbaum et al., 2017), it is usually considered a static property of networks. However, we found the structure of correlated variability to be surprisingly dynamic: the most prominent modes of variability in the network (the PCs) rotated over time, leading to temporal restructuring of functional subnetworks in V1. This may enable V1 populations to conduct different computations at different moments in time during visual processing. Importantly, the speed of these changes in the covariance structure was modulated by cortical feedback in a context-dependent manner. Together, our findings, therefore, indicate a novel role for cortical feedback in temporally organizing the computations in V1.

Feedback projections are thought to be important for perceptual inference (Friston, 2008; Lee and Mumford, 2003; Olshausen, 2013). Several studies have suggested that populations of neurons can perform efficient Bayesian inference using Markov chain Monte Carlo (MCMC) sampling algorithms, where the firing of each neuron represents stochastic samples from the posterior distribution (a probabilistic representation of the beliefs about sensory information given the input) (Buesing et al., 2011; Echeveste et al., 2020). MCMC algorithms produce autocorrelated samples, which allow sampling from high-dimensional distributions but could lead to slow inference. Consequently, the efficiency of these samplers could be modulated by controlling the time constant of their autocorrelation function (Echeveste et al., 2020). LM feedback selectively modulates temporal interdependencies (autocorrelations) in V1 circuits and may thereby play a role in optimizing probabilistic sampling processes according to behavioral demands. Moreover, feedback from higher cortical areas has been proposed to provide expectations and beliefs about the world to earlier processing stages during perceptual inference (Friston, 2008; Keller and Mrsic-Flogel, 2018; Lee and Mumford, 2003; Olshausen, 2013). The rapid changes in the influence of feedback on V1 activity over time may be a hallmark of evolving predictions in a dynamically changing environment.

In summary, we find that cortical areas interact through dynamically rotating communication channels to process and disambiguate sensory signals depending on the behavioral context.

## STAR★Methods

### Key resources table

**Table:** 

REAGENT or RESOURCE	SOURCE	IDENTIFIER
**Bacterial and virus strains**		

AAV1.EF1a.DIO.hChR2(H134R)-eYFP.WPRE.hGH	(Hippenmeyer et al., 2005)	Addgene # 20298

**Chemicals, peptides, and recombinant proteins**		

DiI	Thermo-Fisher	Cat # D3911

**Experimental models: Organisms/strains**		

Mouse: B6;129P2-Pvalbtm1(cre)Arbr/J	The Jackson Laboratory	JAX: 008069

**Software and algorithms**		

MATLAB	Mathworks	https://uk.mathworks.com/products/matlab.html
LabVIEW	National Instruments	https://www.ni.com/en-gb/shop/labview.html
Psychtoolbox 3	(Kleiner et al., 2007)	http://psychtoolbox.org/
Kilosort	(Pachitariu et al., 2016)	https://github.com/MouseLand/Kilosort2
Phy	(Rossant et al., 2016)	https://github.com/cortex-lab/phy
StimServer	(Muir and Kampa, 2014)	https://bitbucket.org/DylanMuir/stimserver
Custom code for experiments	This manuscript	https://doi.org/10.5281/zenodo.6512457
Custom code for data analysis	This manuscript	https://doi.org/10.5281/zenodo.6512262

### Resource availability

#### Lead contact

Further information and requests for resources and reagents should be directed to and will be fulfilled by the lead contact, Sonja B. Hofer (s.hofer@ucl.ac.uk).

#### Materials availability

This study did not generate new unique reagents.

### Experimental model and subject details

All experiments were conducted in accordance with institutional animal welfare guidelines and licensed by the UK Home Office and the Swiss cantonal veterinary office. A total of 20 PV-Cre mice (Hippenmeyer et al., 2005) were used. Electrophysiological recordings and optogenetic manipulations were performed in 4 untrained mice and 16 mice trained in the visual discrimination task (7 and 9 mice with silencing of V1 and LM, respectively, 2 out of 16 mice were excluded due to their task performance, see analysis of behavior). Mice were of either sex and were between 6 and 14 weeks old at the start of the experiment.

### Method details

#### Surgical procedures and virus injection

Prior to surgery, mice were injected with dexamethasone (2–3 mg kg−1) and analgesics (carprofen; 5 mg kg−1). A subgroup of animals was also injected with atropine (0.05–0.1 mg kg−1). General anaesthesia was induced either with a mixture of fentanyl (0.05 mg kg−1), midazolam (5 mg kg−1) and medetomidine (0.5 mg.kg−1), or with isoflurane (1%–4%). A custom headplate was attached to the skull using dental cement (C&B Super Bond) and the skull above the posterior cortex was carefully thinned and sealed with a thin layer of light-cured dental composite (Tetric EvoFlow). Viral injections of AAV1.EF1a.DIO.hChR2(H134R)-eYFP.WPRE.hGH (Addgene 20298) (titre: $2.2\times 10^{12},$ diluted 1:4 in cortex buffer (described below), 70 nl) were made using glass pipettes and a pressure injection system (Picospritzer III, Parker) in the right hemisphere in either V1 or LM. The injection site was identified using intrinsic imaging maps of visual cortical areas (see intrinsic signal imaging) several days prior to the surgery. Some animals were additionally given antibiotic and analgesic drugs (enrofloxacin 5 mg kg−1, buprenorphine 0.1 mg kg−1) at the end of surgery and for 3 days during recovery. Electrophysiological recordings were performed approximately 2-4 weeks after the viral injections.

#### Intrinsic signal imaging

To determine retinotopically matched locations in V1 and LM, mice underwent optical imaging of intrinsic signals (Schuett et al., 2002). This procedure was done a minimum of 3 days after the implantation of a headplate and thinning of the skull (see surgical procedures). On the day of intrinsic imaging, mice were either initially sedated (chlorprothixene, 0.7 mg kg−1), and imaging was carried out either under light isoflurane anaesthesia (0.5–1%) delivered via a nose cone, or imaging was performed in awake, head-fixed mice, free to move on a 20-cm-diameter Styrofoam cylinder.

The visual cortex was illuminated with 700-nm light split from a LED source into two light guides. Imaging was performed with a tandem lens macroscope focused 500 μm below the cortical surface and a bandpass filter centered at 700 nm with 10 nm bandwidth (67905; Edmund Optics). Images were acquired with a rate of 6.25 Hz with a 12-bit CCD camera (1300QF; VDS Vosskühler), a frame grabber (PCI-1422; National Instruments) and custom software written in Labview (National Instruments). The visual stimulus, presented on a display 21 cm away from the left eye, was generated using the open-source Psychophysics Toolbox (Kleiner et al., 2007) based on Matlab (MathWorks) and consisted of a 20° (radius) large square-wave grating, (0.08 cycles/degrees) drifting at 4 Hz in 8 random directions, presented on a grey background for 2 seconds, with a 18 second interstimulus interval alternatively at two positions, at 15° elevation and either 50° or 70° azimuth. Frames in the second following stimulus onset were averaged across 16 grating presentations to generate intrinsic response maps. Response maps to the grating patches at either position were used to identify retinotopically matched locations across areas V1 and LM.

#### Visual discrimination task and visual stimuli

Mice were trained for 2 - 8 weeks to perform a go/no-go task, in which they had to discriminate two static gratings of 45° and -45°orientation. Mice were food restricted throughout the training and electrophysiological recording, with maximum weight loss of 20% of their initial body weight. The restriction started 3 days after the first surgery (headplate implantation) and was interrupted for several days after viral injections, for mice to recover after surgery. The mice were trained for the duration of approximately 1 hour every day. Initially, mice were trained to run head-fixed on a freely rotating Styrofoam cylinder in front of a display (Dell U2715H, 60Hz) placed 24 cm away from their left eye. In later stages of training, when mice were comfortable and used to the paradigm, the cylinder was fixed in place, preventing it from moving. Mice learned within a few days to transition from running to sitting still while performing the task.

Visual stimuli consisted of two 40° diameter large static square-wave gratings of 100% contrast, 0.06 cycles per degree spatial frequency, and either 45° or -45° orientation, presented on a grey background in the center of the monitor. The center of the monitor was placed at approximately 60° azimuth and 15° elevation, and was adjusted later according to the receptive fields of recorded neurons (described below). The luminance of the monitor was 0 $cd/m^{2}$, 16 $cd/m^{2}$, and 32 $cd/m^{2}$ at black, grey and white values, respectively. Gratings were presented for 500ms. The interstimulus interval consisted of a fixed period of 500 ms, plus a random delay with a truncated exponential distribution to avoid extremely long trials (mean: 4-6 seconds, truncation threshold: between 10 and 30 seconds).

6 mice were trained with the 45° grating as the go and the -45° stimulus as the no-go stimulus. For the remaining 10 animals, the identity of go and no-go stimuli was switched. Go and no-go stimuli occurred with equal probability. We did not find any stimulus-specific differences in V1 and LM activity or their interactions, therefore data was pooled across all animals. A reward delivery spout was positioned under the snout of the mice from which a drop of soy milk, or Ensure Plus strawberry drink was delivered in go trials triggered by licking of the spout during a response window of 100 to 550ms (n = 8 animals) or 100 to 650ms (n = 8 animals) after stimulus onset. If mice licked the spout during this time window in response to the go stimulus, trials were classified as hit trials, otherwise as miss trials. In the miss trials, mice received an automatic reward (smaller drop of reward) after the response window. The same time window was used to classify no-go trials into false alarm or correct rejection trials. Licking to the no-go visual stimulus (false alarm) was not punished. Licks were detected with a piezo disc sensor placed under the spout. The detection of licks, reward delivery, and the presentation of visual stimuli were controlled by a MATLAB-based script, StimServer (Muir and Kampa, 2014) (an open-source stimulus sequencing package based on the Psychophysics Toolbox), and using a data acquisition board (PCIe-6321; National Instruments).

#### *In vivo* electrophysiology and optogenetic manipulations

Electrophysiological recordings were performed 2 - 4 weeks after the viral injection. Most mice were trained in the visual discrimination task prior to the recording day. On the day of the recording, mice were anaesthetized with 1%–2% isoflurane, and two 1 mm craniotomies were made above the pre-selected retinotopically matched locations in V1 and LM (see intrinsic signal imaging). Craniotomies were covered with 1.5-2% agarose in cortex buffer, containing (in mM) 125 NaCl, 5 KCl, 10 Glucose monohydrate, 10 HEPES, 2 MgSO4 heptahydrate, 2 CaCl2 adjusted to pH 7.4 with NaOH. A well was built around the two craniotomies, using light-cured dental composite (Tetric EvoFlow), and finally, the well was further sealed with Kwik-Cast sealant (World Precision Instruments).

Mice recovered from surgery for 1-2 h before the recording, and were then head-fixed on a styrofoam cylinder, free to move prior to the start of recording. The Kwik-Cast sealant was removed and a silver wire was placed in the bath for referencing. Two NeuroNexus silicon probes (A2x32-5mm-25-200-177-A64), labelled with DiI, were lowered to 800-1000 μm below the cortical surface using micromanipulators (Sensapex). The electrode positions in V1 and LM were chosen based on the intrinsic map (see intrinsic signal imaging), to target retinotopically matched and therefore anatomically connected locations in the two areas (Marques et al., 2018; Wang and Burkhalter, 2007) (Figure S1A). The electrode positioned in the injection site (in V1 for V1 silencing and in LM for LM silencing) had a 200μm optical fibre (CFMLC52U; Thorlabs) attached with dental cement with its tip 1mm above the electrode tip, placing the fiber tip on the cortical surface once the electrodes were positioned in the cortex.

The craniotomies were then covered with 1.5-2% agarose in cortex buffer. Voltages from 128 channels across two areas were acquired through amplifier boards (RHD2132, Intan Technologies) at 30 kHz per channel, serially digitized and sent to an Open Ephys acquisition board (Siegle et al., 2017) via a SPI interface cable. The wheel was fixed in place (no movement possible), and mice sat quietly on the wheel due to the prior training. Before the start of the behavioral paradigm, retinotopic mapping of the neurons in V1 and LM electrodes was performed, using flashing black and white squares of approximately 20° × 20° on a grey background in 5×4 locations across the monitor. Responses to these stimuli were used to position the monitor, centered on the average receptive field center of recorded neurons.

To silence neuronal activity in either V1 or LM, we optogenetically activated ChR2-expressing parvalbumin-positive neurons using a 473 nm laser (OBIS 473nm LX 75mW; Coherent) or LED (M470F3; Thorlabs) coupled through a patch cable (M73L01; Thorlabs) to the fibre above the area previously injected with the AAV. The light pulse lasted for 150ms. During the first 75 ms, the power was constant at 4 mW, and during the second 75ms, the power was linearly ramped down to zero, in order to prevent rebound spiking. The light pulse was generated using Pulse Pal (open source pulse generator). Optogenetic silencing effectively suppressed neural activity (Median change in the firing rate of V1 neurons was -95.74% when silencing V1, and -93.02% for LM neurons when silencing LM). Propagation of light to the eye was blocked by the cement wall around the craniotomies, as well as black tape shielding the fibre-mating sleeves. Since the fibre tip was far from the eye (> 5 mm) light did not propagate to the retina through the brain (Yizhar et al., 2011). Light was delivered during 8 different time windows during the presentation of the visual stimulus. The onset of light delivery with respect to visual stimulus onset (silencing onset) was randomized from trial to trial. The exact alignment of light onset and visual stimulus was confirmed offline, using recordings of the input pulse to the laser, and the monitor frame update times captured by a photodiode attached to the monitor. These onsets were at 0 ms, 56ms, 123ms, 189ms, 256 ms, 323ms, 390ms and 456 ms after visual stimulus onset (mean across n = 14 animals). We ensured minimal trial-to-trial jitter in laser onset delays (standard deviation of light onset time < 0.3 ms). Control trials without light were interspersed randomly. One session was recorded per mouse, and each session consisted of on average 78 ± 8 (mean ± s.d.) silencing trials for each time window, and 97 ± 16 (mean ± s.d.) control trials (before excluding trials based on the criteria described below).

### Quantification and statistical analysis

#### Analysis of behavior

A behavioral d-prime was calculated for each animal (d’ = Z(hit rate) - Z(false alarm rate), where function Z is the inverse of the standard normal cumulative distribution function (Stanislaw and Todorov, 1999), and only animals with task performance above chance level were included for further analysis (2 animals out of 16 excluded). Chance level was the 99 percentile of the trial-shuffled d-prime distribution, obtained by shuffling the identity of go and no-go trials 5000 times. Average task performance for the animals included in the analysis was d’ = 1.7 ± 0.16 (mean ± s.e.m). Only correct trials (hit and correct rejection trials) were used for subsequent analyses. Moreover, periods of time in the session during which the mice were grooming (continuous prolonged movement detected from the spout readout) were excluded.

#### Electrophysiology data pre-processing

Spikes were sorted with Kilosort (https://github.com/cortex-lab/Kilosort) using procedures previously described (Pachitariu et al., 2016), single units were extracted, and manually curated using phy (https://github.com/cortex-lab/phy). A total of 806 single units in the trained animals (n = 14 mice, 371 units in V1 and 435 units in LM) and 236 single units in untrained animals (n = 4 mice, 146 units in V1 and 90 units in LM) were detected. For all subsequent analysis, the firing rate in time windows with an average firing rate below a threshold was set to nan. This was done in order to prevent dividing by values close to zero. The threshold was chosen as the value at which the 95% confidence interval of the firing rate exceeded zero (2.5 Hz). Results were not significantly different when neuronal activity was not thresholded.

#### Receptive field calculation

Receptive fields were calculated by fitting a two-dimensional Gaussian distribution to the responses of neurons to the flashing black and white square stimuli. The center of the receptive field was calculated as the peak of the Gaussian fit. Since receptive fields were calculated from square stimuli on a flat screen, the receptive field locations in visual degrees are approximate. However, this does not affect the relative comparisons between the regression models in V1 and LM (described below).

#### Quantifying the effect of silencing

The change caused by optogenetic manipulation to the firing rates of individual neurons was quantified as $100\times \left(R_{s}\;-\;R_{c}\right)/R_{c}$ for each silencing time window, where $R_{s}$ denotes the cell’s spike count during the 150ms silencing time window, and $R_{c}$ denotes the spike count in the corresponding time window in control trials. Significant effects were determined based on exceeding the 95% confidence interval of percentage differences in the firing rate of bootstrapped control trials. To determine the number of significantly affected time windows per cell, the significance levels were adjusted using Bonferroni correction for 8 comparisons.

#### Multivariate regression model

We used a generalized linear regression model in order to examine if silencing effects could be explained by physiological and anatomical properties of target neurons such as cortical depth. We predicted the activity of LM neurons (target area) during optogenetic silencing of V1 (source), from their own activity in control trials (in the corresponding time bin) and the depth of LM neurons in the cortex (Figure S2):

$$R_{s}\;∼N\left(\mu ,\;\sigma \right)$$

$$\mu \;=\;\left(d\;\beta^{d}\;+\;d^{2}\;\beta_{2}^{d}\right)\;+\;\left(R_{c}\;\beta^{c}\;+\;R_{c}^{2}\;\beta_{2}^{c}\right)\;+\;dR_{c}\;\beta^{i}\;+\beta^{0}\;$$

Where $R_{s}$ denotes the spike count during the 150 ms source-silencing time window, modeled with a Gaussian distribution with the mean and standard deviation of μand σ. $R_{c}$ denotes the spike count in the corresponding window in control trials, d the depth of the target neuron soma, and β the model coefficients. Cortical depth was determined based on current source density analysis (CSD), with zero denoting the center of the initial current sink (which roughly corresponds to thalamic input in layer 4 in V1). The model was fit with the iteratively reweighted least squares algorithm, using the fitglm function from MATLAB. The performance of the model was assessed using 20-fold cross validation. For each set of training and test sets, the model parameters were fit using the training set, and the log likelihood of the test set was calculated ($log_{2\;}l$). We also used the same procedure to calculate the log likelihood of a null model that only contained information about the LM cell’s control trial activity ($\mu_{null}\;=\;R_{c}\;\beta^{c}\;+\;R_{c}^{2}\;\beta_{2}^{c}\;+\beta^{0}$ ). This gives a null likelihood value ($log_{2\;}l_{0}$).

We then measured model performance as the degree to which the full model outperforms the null model by calculating the trial-averaged excess log likelihood of the full model compared to the null model. This would capture any dependence of the silencing effect on the depth of LM neurons, that is not solely due to the relationship between activity levels and cortical depth.

Model performance [bits/trial] = $\left(log_{2\;}l\;-\;log_{2}\;l_{0}\right)/N$

Where N is the number of observations in the test dataset. Model performance was calculated for each of the 20 test sets (observations) separately. Above-chance performance was determined by a one-sided Wilcoxon signed-rank test. We constructed similar, separate models to quantify the influence of other cell properties, such as relative receptive field position and average firing rate over the session. The relative receptive field positions were the distance of the cell's spatial receptive field center to the retinotopic location of silencing in the source area, which was calculated as the average receptive field location of the recorded neurons from an electrode positioned in the center of AAV-flex-ChR2 virus expression in the source area. We repeated the same procedures for predicting the activity of V1 neurons during optogenetic silencing of LM.

The same regression model and procedures were used to quantify to what degree neurons’ response selectivity to the visual stimuli or to behavioral choice explained the effect that silencing the source area had on them (Figure S3). Selectivity index for behavioral choice was calculated per neuron as $\left(\left(R_{H}\;-\;R_{M})/(R_{H}\;+\;R_{M})+(R_{F}\;-\;R_{C})/(R_{F}\;+\;R_{C}\right)\right)/2$, where $R_{H},\;R_{M},\;R_{F},\;R_{C}$ denote the average firing rate of the neuron during the visual stimulus presentation (500 ms) in hit (go stimulus presented, animal licks), miss (go stimulus presented, animal does not lick), false alarm (no-go stimulus presented, animal licks), and correct rejection trials (no-go stimulus presented animal does not lick) respectively. Selectivity index for the visual stimulus was calculated as $\left|(r_{H}\;-\;r_{C})/(r_{H}\;+\;r_{C})\right|$ where $r_{H\;}$and $r_{C\;}$denote the firing rate of the neuron during the first 150 ms (before the motor movement) of hit and correct rejection trials respectively. The identity of the preferred stimulus, indicating which of the go or no-go stimulus the neuron preferred, was calculated as $sign\left(\left(r_{H}\;-\;r_{C})/(r_{H}\;+\;r_{C}\right)\right)$. As a control, selectivity index for the visual stimulus was also calculated using an alternative method, from the choice-matched trials, as $\left|((R_{H}\;-\;R_{F})/(R_{H}\;+\;R_{F})+(R_{M}\;-\;R_{C})/(R_{M}\;+\;R_{C}))/2\right|$.

#### Effect of silencing on stimulus decoding and behavioral performance

To examine the effect of silencing one area on stimulus coding in the other area, we performed the following analyses. Silencing trials with onset times earlier than 100 ms after stimulus onset, were classified as early (before any measured behavioral response), and the rest as late. We calculated the performance of a linear decoder on classifying correct go and correct no-go trials using either the activity of neurons in V1 with and without LM silencing, or using the activity of LM neurons with and without V1 silencing. The activity of each neuron was calculated as its average firing rate in the 80 ms time window following the silencing onset during silencing trials, and the corresponding temporal window during control trials. Classification was performed using regularized linear discriminant analysis (LDA), where the regularization parameter was chosen separately for each animal as the value maximizing the decoding performance on test trials. The classifier was trained separately for each silencing time window, on 80% of the control trials. The control decoding performance was assessed on the remainder of the control trials, and the silencing decoding performance was calculated from the silencing trials (using the same classifier trained on control trials). Dividing control trials into a training and test set was performed 20 times and the decoding performance was calculated as the average across the 20 test sets. For these analyses, only animals with at least 10 control trials (n = 7 of 7 and n = 6 of 7 mice for V1 and LM silencing, respectively) were included, and an equal number of go and no-go trials were used to train the classifier. As a control, we also calculated the neuron- and trial-averaged (over both go and no-go trials) spiking activity of neurons in the target area (Figures S5C and S5F). The average activity was calculated using all control or all silencing trials (separately), using the same time windows used for stimulus decoding above.

To assess the effect of silencing V1 or LM on the animals’ behavioral performance, we calculated reaction times and behavioral d-primes, and compared effects of silencing in early or late silencing windows as above (starting earlier or later than 100 ms after visual stimulus onset). The same animals used for the decoding analysis above (n = 7 of 7 and n = 6 of 7 mice for V1 and LM silencing, respectively) were included here. Reaction time was defined as the time to first lick after stimulus onset, using only correct go trials, and the percentage of change compared to the average reaction time was reported for each animal: $100\;\times \;(rt-mean\left(rt\right))/mean\left(rt\right)$, where rt denotes the reaction time. In order to measure the effect of silencing only on behavioral responses that occur after the silencing onset, we included for each silencing time window only trials in which licking started after the time window’s onset. This was done separately for both control and silencing trials at each time window and was called the onset-corrected reaction time. Note that this correction results in different reaction times for different time windows, and reaction times of late time windows are therefore not indicative of the true reaction time of the animals. However, this does not affect the comparison between the silencing and control trials. We also reported the percentage of change in the raw reaction times, without onset correction (Figures S5A and S5D).

Changes in behavioral d-primes were reported as the percentage of change compared to the average d-prime per animal $100\;\times \;(dp-mean\left(dp\right))/mean\left(dp\right)$, where dp denotes d-prime. As with the reaction times, dp was calculated for silencing and control trials, only for trials with licking onset after onset of the silencing time window or the corresponding time window in control trials. Note that this onset correction results in different d-primes for late versus early silencing time windows, however comparisons were only made between equivalent time windows in control and silencing trials. We also reproduced the same plots using the raw d-prime without onset correction (Figures S5B and S5E).

#### Causal communication direction

We characterized the influence of the source area on target neurons on the population level. For a population of n simultaneously recorded neurons in the target area, we found an n×1vector, in the n dimensional activity space that maximally separated the activity vectors in the control and source-silencing trials. This was done separately for each silencing time window and stimulus type (go/ no-go). The source-silencing activity vectors corresponded to the population firing rate of the n neurons in the 150 ms in which the source area was optogenetically silenced, and the control activity vectors were the firing rate of the same population in the corresponding 150 ms time window in control trials. In order to find the direction maximally separating the control and source-silencing trials, we used regularized linear discriminant analysis (LDA). Communication direction (CD) was defined as the unit-length normal vector to the decision boundary hyperplane.

$$CD={\hat{\Sigma_{\gamma }}}^{\;-1}\left(\mu_{c}\;-\mu_{s}\right)$$

$$\hat{\Sigma_{\gamma }}\;=\left(1\;-\;\gamma \right)\;\hat{\Sigma }\;+\;\gamma \times diag\left(\hat{\Sigma }\right)$$

Where $\hat{\Sigma }$ denotes the pooled covariance estimate, $\mu_{c},\;\mu_{s\;}$correspond to the mean activity vectors, and γ is the regularization parameter. In addition to γ, we used another parameter for each classifier. Any coefficients in CD with a magnitude smaller than a threshold, δ, was set to zero, eliminating the corresponding neuron from the discriminant direction.

The two parameters of discriminant classifiers (γ,δ) were fit separately for each animal by minimizing the test error in a ten-fold cross validation. Excluding δ did not change results significantly. The classifier performance for the LDA classifiers in trained animals are shown in Figures S6A–S6F. All included animals had above chance classification performance.

For the above analysis, only animals with at least 10 control trials (n = 7 of 7 and n = 6 of 7 mice for V1 and LM silencing, respectively), and only visually responsive cells were included. Neurons had to spike at least once (on average) during the 500 ms stimulus duration in response to the go or no-go stimulus, and there needed to be a significant difference (p = 0.05, two-sided Wilcoxon signed-rank test) between the spike count during the stimulus and a 500 ms window before stimulus onset for at least one of the stimuli. An equal number of go and no-go trials were used for calculating the communication direction at each time window.

The similarity of two directions (cosine angle) was captured by their dot product $cos\left(\alpha_{i,j}\right)=\left(CD_{i}.\;CD_{j}\right)/\left(\left|CD_{i}\right|.\left|CD_{j}\right|\right)\;$, represented in the ith row and jth column of the cosine similarity matrix. In order for this measure to reflect trial-to-trial stability of the communication directions, we used a cross-validated similarity measure. We randomly split the control trials in half, one half was used to calculate $CD_{i}$ and the other, $CD_{j}$. This was then repeated in reverse, and the resulting dot products were averaged. The random partitioning of trials was done 100 times, and the average dot products were reported in the cosine similarity matrices. This method accounts for trial-to-trial variability and summarizes robustness of cosine similarity to trial-to-trial noise. Deviation from 1 of the diagonal elements in the cosine similarity matrix (cosine similarity at lag 0) reflect the trial to trial variability of communication directions at each time window.

Comparing differences in average activity vectors (not normalized to covariance), instead of regularized LDA vectors gave similar results (Figures S8B–S8E). However, we used regularized LDA in order to find the mode of activity which would most reliably specify the difference between control and silencing trials across trials.

In order to track the similarity of trial-averaged population activity over time, we defined activity directions at each time window as the n×1unit length vector specifying the coordinates of trial-average population activity. To calculate the activity directions, we used 65 ms time windows (instead of the full 150 ms time windows used elsewhere), in order to avoid temporal overlap between successive time points. Using 150 ms windows for activity directions resulted in even slower changes over time (data not shown). The activity directions were calculated from V1 and LM activity in control trials, pooled across all animals included in the LM silencing or V1 silencing experiments. The similarity of activity directions was assessed using cross-validated cosine similarity as explained above. Similarity in activity directions implies that different neurons undergo similar temporal fluctuations of activity, leading to a change only in the magnitude of population activity, and not its direction over time (Figure S1C). For comparing how fast similarity of activity and communication directions decay over time, we used the initial slope (slopes between lag 0 and lag 1) of the decay over time lags of pairwise similarities, (decay slope of similarity between adjacent time windows). This initial decay slope, as opposed to exponential decay time constant, does not assume an underlying exponential decay, and can be used as a general measure. We chose this measure since the similarity of activity directions decays linearly rather than exponentially (different to the similarity of communication directions) over time.

We used raw firing rates of neurons for calculating the activity or communication directions described above, reflecting the neurons’ overall activity, and not only their response to the visual stimuli. However, we also repeated the analyses using baseline-subtracted activity, calculated by subtracting neurons’ average firing rate in the 500 ms time window preceding the visual stimulus from the responses during visual stimulus presentation (Figure S8F). Using baseline-subtracted visual responses or raw firing rates to calculate activity and communication directions yielded similar results (Figure S8F).

#### Sub-selection of cells in the source area

To rule out the possibility that the fast changes in the similarity of communication directions over time arise from changes in the activity direction of a select subnetwork of neurons in the source area (e.g., projection neurons) with particularly fast dynamics, we examined the activity directions of the subgroup of source neurons with the most dynamic firing rate compared to the average population. In order to identify a subpopulation of neurons in the source area with the fastest decay in their similarity of activity vectors, we z-scored the activity of all simultaneously recorded source neurons at each time window separately. Then, we selected 20% of cells with the highest standard deviation over time in their z-scored activity. This procedure leads to selecting subpopulations that have the highest deviation from the average population activity over time, and would therefore have the fastest change in their activity vector similarity over time. In order to compare the cosine similarity of activity directions derived from this subpopulation to those of the communication directions, we re-calculated the communication directions from a randomly selected subpopulation of 20% of target neurons (averaged over 100 repeats), This sub-selection of target neurons was done in order to make the maximum dimensionality of communication directions comparable to those of the source area activity directions.

#### Simulation of time invariant inter-areal influences

In order to simulate time-invariant influence of areas on each other, we generated a dataset in which the activity of each target neuron during source-silencing was simulated based on its control activity and assuming similar source-silencing effects during all time windows. For this, for each target neuron, the source-silencing effect of one of the 8 silencing time windows ($E^{T}$) was selected randomly and used for all time windows as the cell’s time-invariant silencing effect (E), with the randomization repeated 100 times. Next, for each silencing time-window, the source-silencing spike count was simulated by first generating a Poisson sample with the mean of control trial spike counts in the same time window ( $\hat{R_{c}^{T}}$), and then, subjecting the activity of each simulated trial to the time-invariant silencing effect (E), to get the simulated source-silencing spike counts ($\hat{R_{s}^{T}}$):

$$E^{T}\;=\;100\times \left({R_{s}}^{T}\;-\;{R_{c}}^{T}\right)/{R_{c}}^{T}$$

$$E\;=\;E{\;}^{T_{rand}}$$

$$\hat{R_{s}^{T}}\;=\;\left(1+E/100\right)\times \;\hat{R_{c}^{T}}$$

T∈{1,..,8}

Where ${R_{s}}^{T}$and ${R_{c}}^{T}$ are the spike count in source-silencing and control trials during the silencing time window T. By generating spike counts using a Poisson distribution, the trial-to-trial variability of target neuron activity is preserved, while the underlying source-silencing effect is assumed to be time-invariant. Since the time-invariant source-silencing effect on each target neuron is selected randomly based on its own effects, over 100 repeats, the time-averaged source-silencing effects are similar to the experimental dataset, and the overall effect size of source-silencing was preserved in the simulated dataset. Importantly, since the simulations are based on the control firing rate of target neurons in each time window which contain input from the source area, we account for the compound trial-to-trial variability in target area activity and source area input.

#### Magnitude of population-level influences

To determine the magnitude of the feedforward influence of V1 on LM population activity, we calculated the Bhattacharyya distance - a measure of the distance between two distributions in high-dimensional space - between LM population activity in control and V1-silencing trials, assuming multivariate normal distributions. The covariance was assumed to be similar in the two conditions and was estimated by pooled covariance from both control and silencing trials (similar to the LDA, see above). The distances were calculated separately for each V1-silencing time window. We calculated the noise level as the average Bhattacharyya distance between bootstrapped samples of population activity in control trials. This noise level describes the distance expected in the absence of any silencing effect, caused by trial-to-trial noise in control trials. The magnitude of the feedback influence of LM on V1 population activity was calculated similarly, using V1 population activity in control and LM-silencing trials.

#### Principal component analysis

The principal components (PCs) of V1 activity were calculated separately for the population activity during each 150 ms time window, either in the control or LM silencing trials. PCs were calculated in each time window separately for go and no-go trials, and for control and silencing conditions, with an equal number of trials used across the 4 conditions. In order to calculate the similarity of PC directions over time, we used the absolute value of cross-validated similarity as described above (see causal communication direction section), where only half of the trials were used to calculate PCs at each time window. To calculate the total variance and eigenspectra, we used all included trials at each time window to calculate the variance along each principal component. Similarly to the communication direction analysis, only animals with at least 10 control trials, and only visually responsive cells were included in the analysis (for responsiveness criteria see causal communication direction section). To quantify the decay of PC similarity across time lags without explicitly assuming an exponential decay, we used the decay slope described above (see causal communication direction section).

#### Exponential fits

For comparing time constants of exponentially decaying functions, the decay of cosine similarity over time lags was fit by an exponential decay with an offset:

y=A(exp(−t/τ)+B)

Where t is the time lag between two silencing onset times. Data was fit using nonlinear least-squares fitting via the trust-region algorithm (through MATLAB fit function). Confidence intervals of the fit parameters were obtained using a 100-times bootstrap. To assess the significance of the differences between the decay time constants of communication during go and no-go stimuli, we used a permutation test. To do this, we calculated the distribution of time constant differences under the null hypothesis. This null distribution was generated from exponential fits on data with go and no-go trial labels exchanged randomly (1000 randomized permutations).

#### Autocorrelations and pairwise noise correlations

To calculate the temporal autocorrelation of spike counts, we divided the stimulus duration into successive time bins of 65 ms duration. We then calculated across-trial Pearson’s correlation coefficient between the spike counts at any two time bins and plotted against the time lag between the two time bins. This was done separately for each neuron. This method subtracts the across-trial mean spike counts, therefore the spike-count autocorrelations don’t explicitly depend on mean firing rates at different time bins.

Noise-correlation strength was calculated for each pair of neurons in V1 as the across-trial Pearson’s correlation coefficient between the spike counts of the two neurons at a given time bin, using similar time bins as for the autocorrelation analysis. These correlation coefficients were plotted as a function of the average spike count of the pair in the same time bin and values calculated from different time bins were then pooled. For calculating autocorrelations and pairwise noise correlations, similarly to the communication direction analysis, only animals with at least 10 control trials, and only visually responsive cells were included in the analysis (for responsiveness criteria see causal communication direction section), and an equal number of trials was used across go and no-go trials.

#### Statistics

We used two-sided Wilcoxon rank-sum tests for independent group comparisons, and two-sided Wilcoxon signed-rank tests for paired tests, unless otherwise stated. Raw p-values are reported throughout the manuscript, significance thresholds were adjusted for multiple comparisons using Bonferroni correction, as indicated in the legends. Tests were performed using MATLAB. The mean and the standard error of the mean, or 95% confidence intervals were used for displayed purposes, as stated in the figure legends. No statistical methods were used to pre-determine experimental sample sizes in experiments; sample sizes were based on what is conventional for the field and previous literature. Mice were randomly assigned to groups for being trained on 45° or -45° oriented grating as the go stimulus. Blinding to experimental condition was not applicable for this study.

## Data Availability

• Data reported in this paper will be shared by the lead contact upon request. • Software used for the visual discrimination experiment (https://github.com/mitrajz/behavior_code) and analysis code (https://github.com/mitrajz/long_range_com) have been deposited on Github and Zenodo and are publicly available as of the date of publication. DOIs are listed in the key resources table. • Any additional information required to reanalyze the data reported in this paper is available from the Lead Contact upon request.

## Supplemental information

Document S1. Figures S1–S9

Document S2. Article plus supplemental information
