# Supplementary material for: Dynamic causal communication channels between neocortical areas
Source: Neuron. 2022 Aug 3;110(15):2470–2483.e7. doi: 10.1016/j.neuron.2022.05.011 (PMC9616801; doi:10.1016/j.neuron.2022.05.011)
Supplement: Document S1. Figures S1–S9 [file mmc1.pdf]

Neuron, Volume *110*

Supplemental information

Dynamic causal communication channels between neocortical areas

Mitra Javadzadeh and Sonja B. Hofer

Figure S1

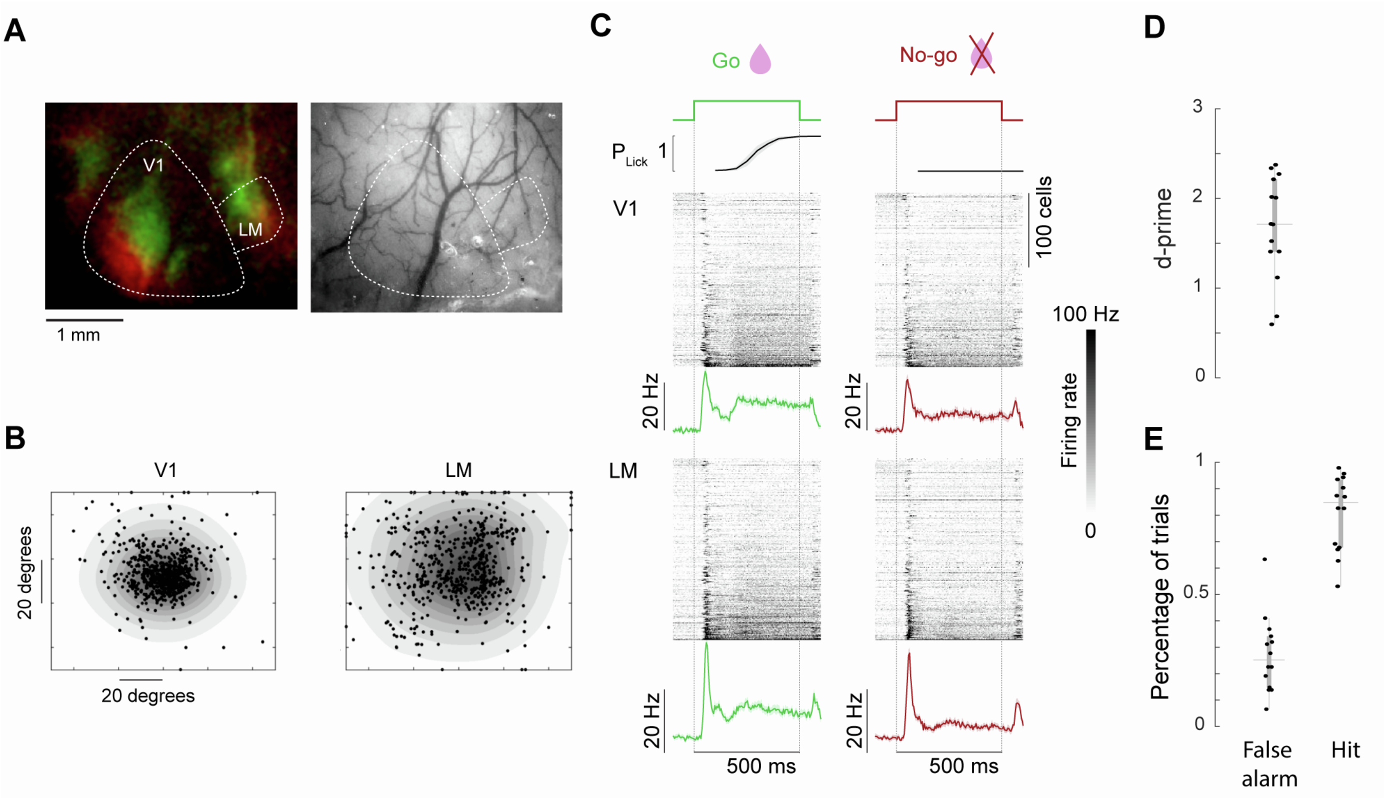

Figure S1. Targeting retinotopically matched regions in V1 and LM using intrinsic signal imaging. Related to Figure 1 and STAR Methods.

(A) Left: example intrinsic imaging map used to target V1 and higher visual lateromedial area (LM). Intrinsic responses evoked by two spatially separated visual stimuli (see STAR Methods) are color-coded in green and red. Right: surface blood vessel pattern corresponding to the intrinsic imaging map on the left. Approximate borders of V1 and LM are outlined.

(B) Receptive field centers of recorded neurons in V1 (left) and LM (right) (n = 16 animals) overlaid on the average receptive field of all neurons, obtained by individual 2D Gaussian fits.

(C) Top, cumulative lick probability over time from stimulus onset (averaged over trials and mice, n = 14 mice) in correct go (left) and correct no-go (right) trials. Shading depicts 95% confidence interval of the mean. Middle and bottom, spiking activity of neurons in V1 (middle) and LM (bottom) in response to the go (green, left), and the no-go (red, right) stimulus. Each row in the heat plots denotes the average firing rate of one neuron. The traces below are average peri-stimulus time histograms (PSTHs) of all recorded visually responsive neurons, binned at 20 ms.

(D) Task performance (behavioral d-prime, see STAR Methods) of all animals included in the analyses (n = 14). The criterium for inclusion in the analyses was task performance above chance level, calculated as the 99 percentile of the trial-shuffled d-prime distribution (see STAR Methods).

(E) Percentage of false alarm and hit trials for all animals included in analyses (n = 14).

Figure S2

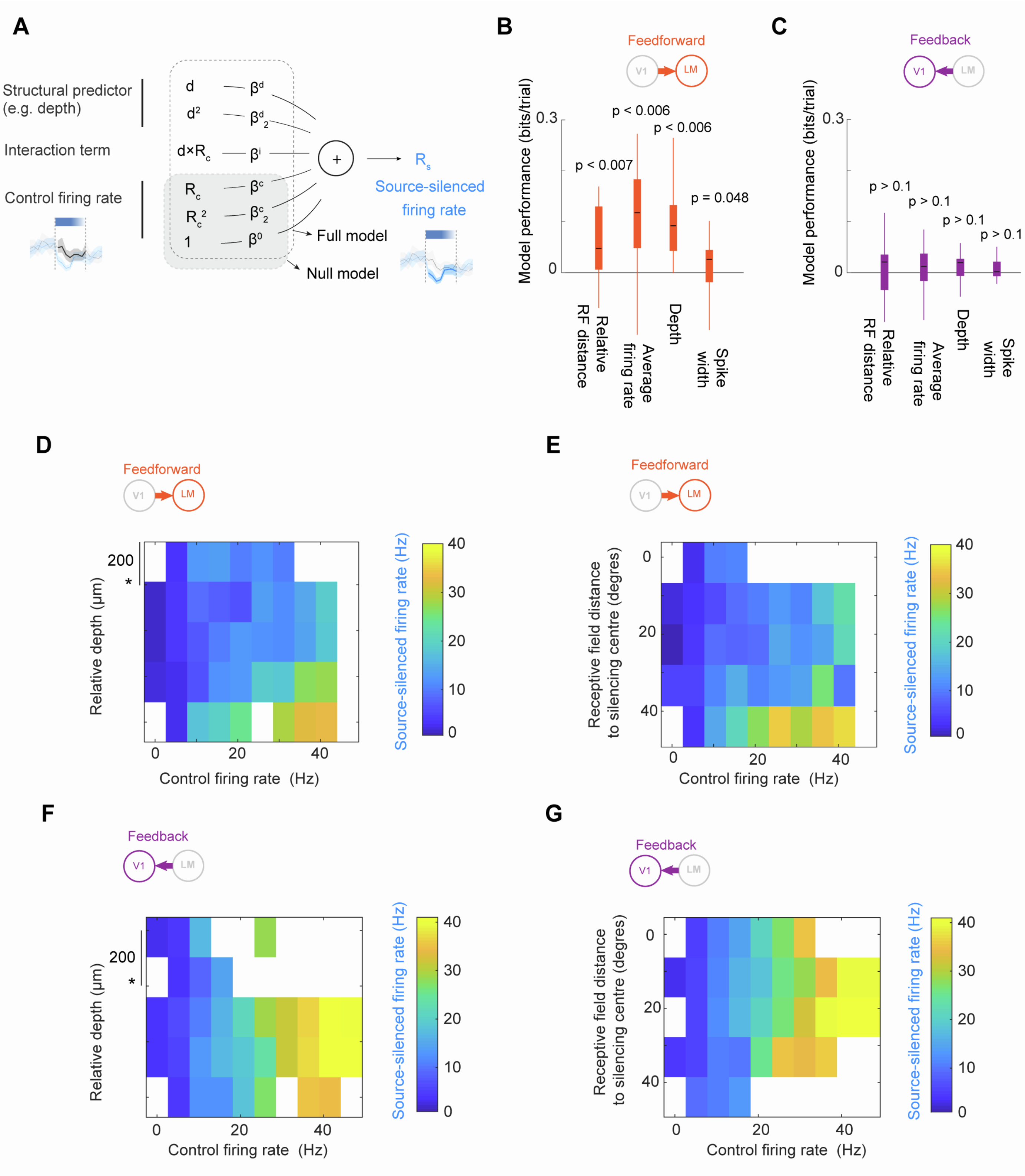

A
Structural predictor
(e.g. depth)
Interaction term
Control firing rate
Full model
Null model
Source-silenced
firing rate
B
Feedforward
V1
LM
Model performance (bits/trial)
0.3
0
p < 0.007
p < 0.006
p < 0.006
p = 0.048
Relative
RF distance
Average
firing rate
Depth
Spike
width
C
Feedback
p > 0.1
p > 0.1
p > 0.1
p > 0.1
D
E
F
G
Relative depth (µm)
200
Control firing rate (Hz)
Source-silenced firing rate (Hz)
Receptive field distance
to silencing centre (degrees)

Figure S2. Dependence of firing rates during source silencing on the properties of target neurons. Related to Figure 1 and STAR Methods.

(A) Regression model predicting the activity of target neurons during optogenetic silencing of the source area, from a combination of the neurons' activity in control trials in the same time window and the structural predictor of interest (e.g. cortical depth). The null model contains information only about the control trial activity. Models were fit separately for the following predictors: distance of spatial receptive field center from the retinotopic location of the center of optogenetic silencing in the source area, average firing rate during the session, depth of neuron in the cortex, and the width of action potential waveforms.

(B) The cross-validated performance of each model predicting the influence of V1 silencing on LM cells (feedforward influence), as the degree to which the model outperforms the null model (in bits/trials). Y axis indicates the log likelihood ratio (full model to null model) of the test observations in each cross-validation fold (n=20), normalized to the number of observations. These values would capture any dependence of the influence of V1 on LM neurons' activity on the particular predictor (indicated on the x axis) that is not explained by the modulation of firing rates by the predictor in control conditions. Values significantly above zero (p-value < 0.05, Bonferroni correction, one-sided Wilcoxon signed-rank test) indicate above-chance prediction power of the model.

(C) As in (B) but for predicting the firing rate of V1 neurons during LM silencing.

(D) Firing rate of LM neurons during V1 silencing (indicated by color code) as a function of their firing rate in control conditions without optogenetic manipulation (control firing rate, x axis) and their depth in the cortex (y axis). Modulation in the vertical axis (for a given control firing rate) indicates the effect of depth on the neurons' activity in the absence of V1 input. The star (*) denotes the center of the initial current sink detected from current source density analysis (in V1 this corresponds approximately to layer 4).

(E) Firing rate of LM neurons during V1 silencing as a function of their control firing rate (x axis) and the distance of their receptive field center from the retinotopic location of the center of optogenetic manipulation in V1 (y axis). Modulation in the vertical axis (for a given control firing rate) indicates the effect of relative receptive field position on the neurons' activity in the absence of V1 input.

(F) As in (D) but for the firing rate of V1 neurons during LM silencing.

(G) As in (E) but for the firing rate of V1 neurons during LM silencing.

Figure S3

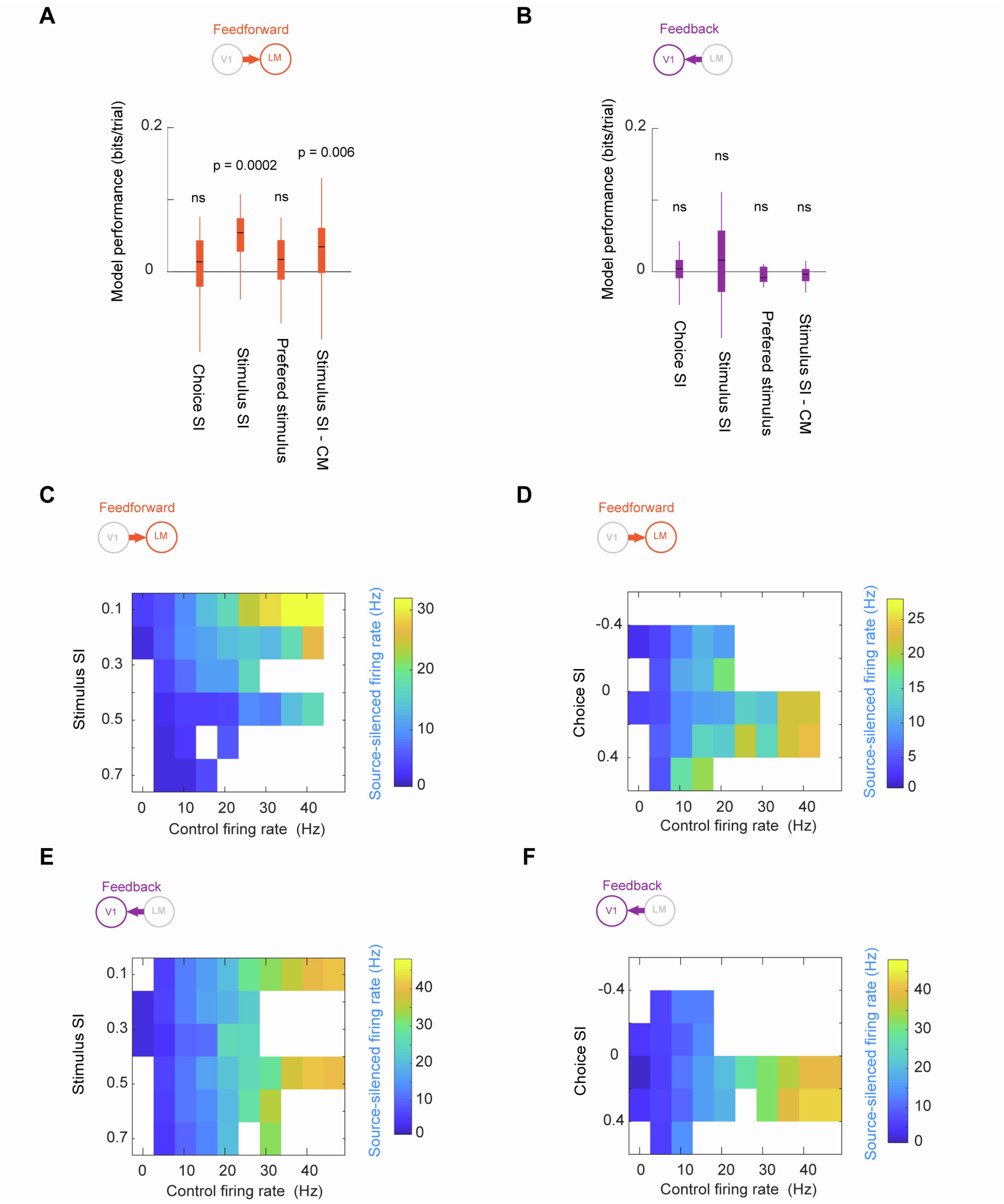
A
Feedforward
V1
LM
Model performance (bits/trial)
0.2
0
ns
p = 0.0002
ns
p = 0.006
Choice SI
Stimulus SI
Prefered stimulus
Stimulus SI - CM
B
Feedback
V1
LM
Model performance (bits/trial)
0.2
0
ns
ns
ns
ns
Choice SI
Stimulus SI
Prefered stimulus
Stimulus SI - CM
C
Feedforward
V1
LM
Stimulus SI
0.1
0.3
0.5
0.7
0
10
20
30
40
Control firing rate (Hz)
Source-silenced firing rate (Hz)
30
20
10
0
D
Feedforward
V1
LM
Choice SI
-0.4
0
0.4
0
10
20
30
40
Control firing rate (Hz)
Source-silenced firing rate (Hz)
25
20
15
10
5
0
E
Feedback
V1
LM
Stimulus SI
0.1
0.3
0.5
0.7
0
10
20
30
40
Control firing rate (Hz)
Source-silenced firing rate (Hz)
40
30
20
10
0
F
Feedback
V1
LM
Choice SI
-0.4
0
0.4
0
10
20
30
40
Control firing rate (Hz)
Source-silenced firing rate (Hz)
40
30
20
10
0

Figure S3. Dependence of firing rates during source silencing on the response tuning of target neurons. Related to Figure 1 and STAR Methods.

(A) As in **Figure S2B,** cross-validated performance of the regression model predicting the influence of V1 silencing on LM cells (feedforward influence), but with different predictors. Choice SI: Selectivity index for behavioral choice-related responses (see STAR Methods), Stimulus SI: Selectivity index for responses to the task-relevant visual stimuli (go and no-go, see STAR Methods), Preferred stimulus: The identity of the preferred stimulus (go or no-go, see STAR Methods), Stimulus SI – CM: Selectivity index for responses to go and no-go stimuli calculated from choice-matched trials (see STAR Methods).

(B) As in (A) but for predicting the firing rate of V1 neurons during LM silencing.

(C) Firing rate of LM neurons during V1 silencing (indicated by color code) as a function of their firing rate in control trials without optogenetic manipulation (control firing rate, x axis) and their stimulus selectivity index (y axis, difference in response to go and no-go stimuli). Modulation in the vertical axis (for a given control firing rate) indicates the effect of stimulus selectivity index on the neurons' activity in the absence of V1 input.

(D) Firing rate of LM neurons during V1 silencing (indicated by color code) as a function of their firing rate in control trials without optogenetic manipulation (control firing rate, x axis) and their behavioral choice selectivity index (y axis, difference in activity in trials with and without licking response). Modulation in the vertical axis (for a given control firing rate) indicates the effect of choice selectivity index on the neurons' activity in the absence of V1 input.

(E) As in (C) but for the firing rate of V1 neurons during LM silencing.

(F) As in (D) but for the firing rate of V1 neurons during LM silencing.

Figure S4

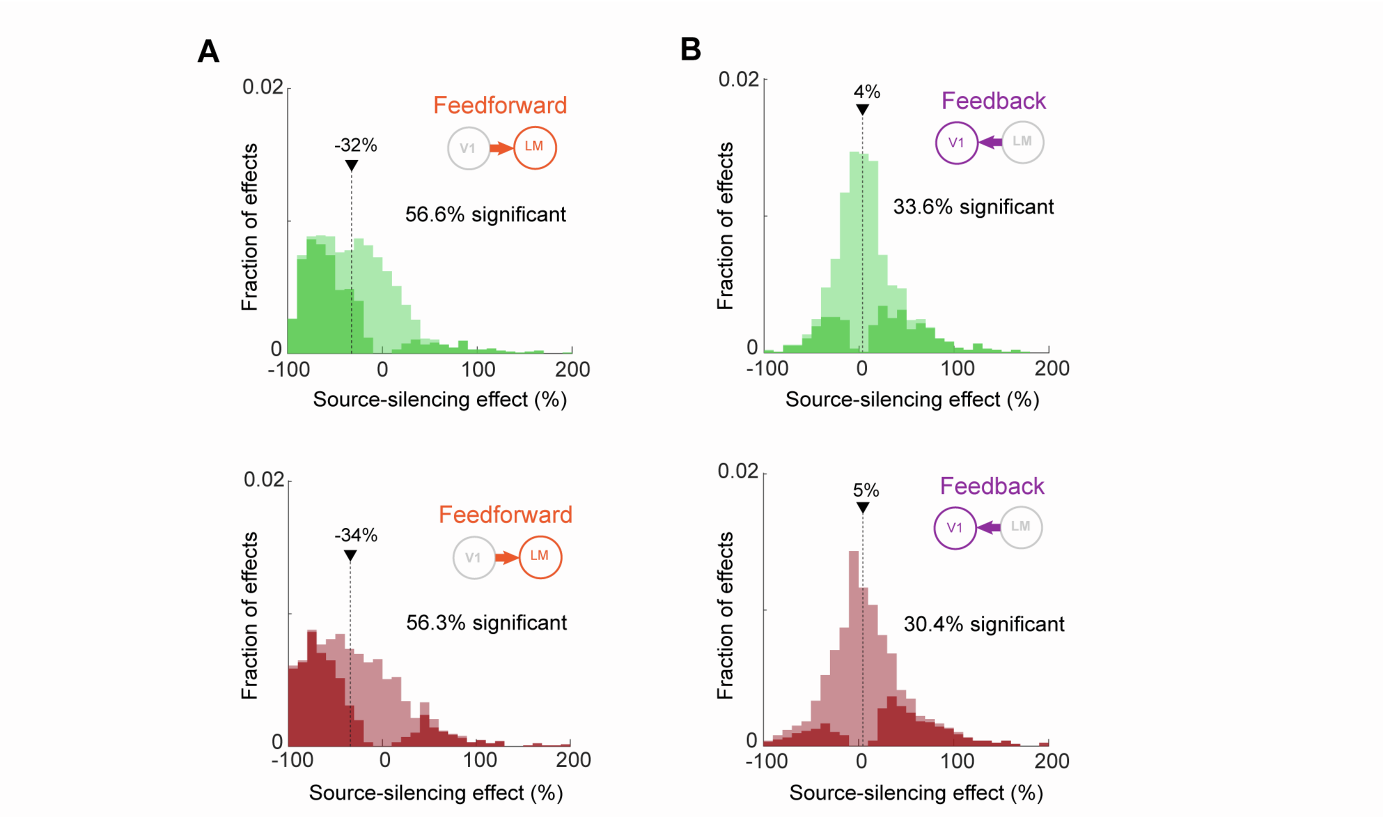

Figure S4. Distribution of silencing effects in Go and No-go trials. Related to Figure 1.

(A) Distribution of the effect of silencing V1 on individual LM neurons (feedforward influence) in all silencing time windows during go trials (green, top) and no-go trials (red, bottom). Significant effects shown in bright colors. The arrow denotes the median of the distribution. Go vs no-go p = 0.87, two-sided Wilcoxon rank-sum test.

(B) Distribution of the effect of silencing LM on individual V1 neurons (feedback influence) in all silencing time windows during go trials (green, top) and no-go trials (red, bottom). Significant effects shown in bright colors. The arrow denotes the median of the distribution. Go vs no-go p = 0.78, two-sided Wilcoxon rank-sum test.

Figure S5

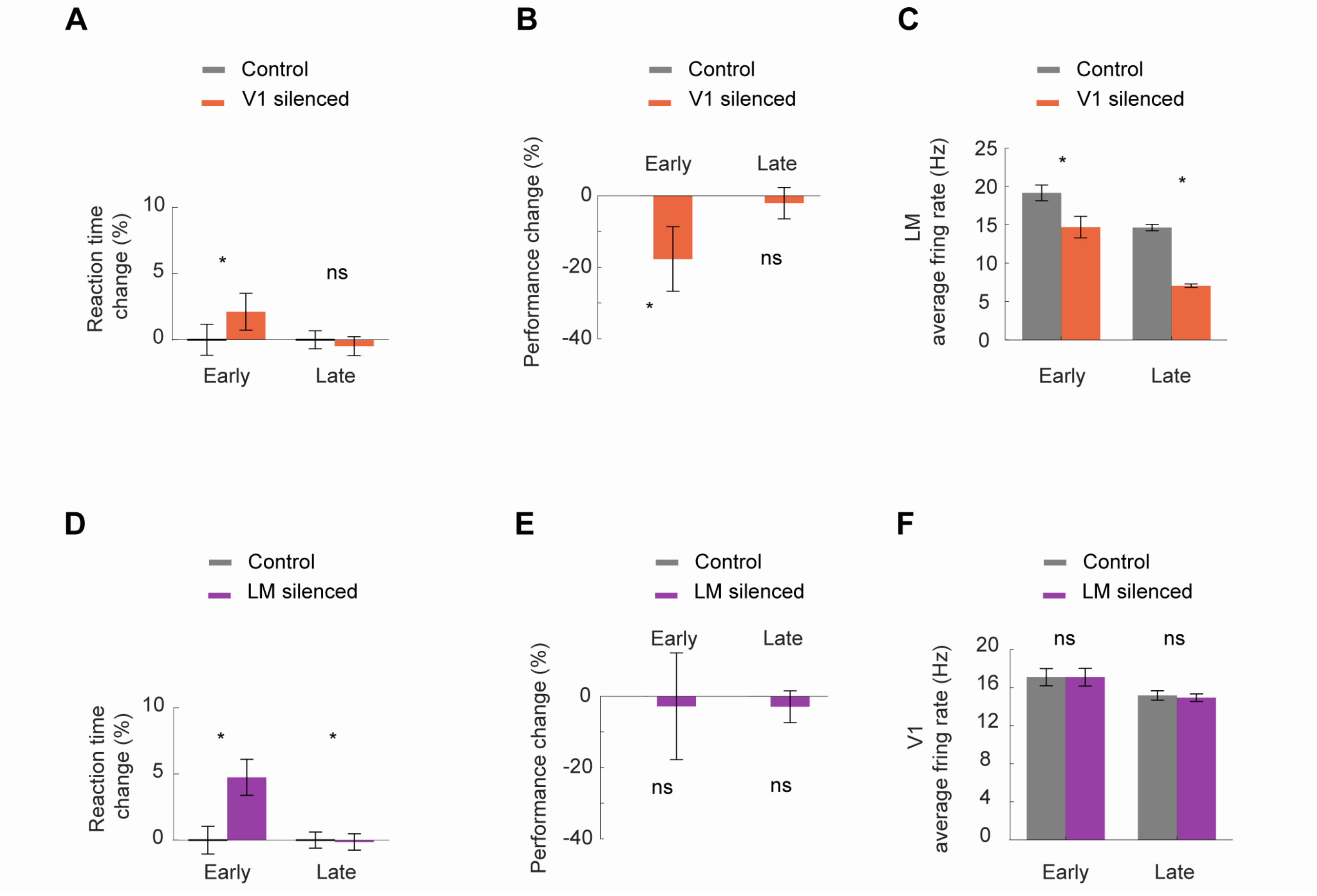

Figure S5. Controls for the behavioral effects of optogenetic silencing of V1 or LM. Related to Figure 2.

(A) As in **Figure 2A**, but showing percentage of change in raw reaction times instead of onset-corrected reaction times, in control (grey) and V1-silencing (orange) trials. P-values from two-sided Wilcoxon rank-sum test ($p = 0.049$ for early silencing and $p = 0.629$ for late silencing).

(B) As in **Figure 2B**, but showing percentage of change in the raw behavioral d-primes instead of the onset-corrected d-primes, in control (grey) and V1-silencing (orange) trials. P-values are from Wilcoxon two-sided signed-rank test ($p = 0.035$ for early silencing and $p = 0.512$ for late silencing).

(C) Average firing rate of LM neurons (across trials and neurons), in the time windows corresponding to those used in **Figure 2C,** in control (grey) and V1-silencing (orange) trials. P-values are from Wilcoxon two-sided signed-rank test ($p = 0.011$ for early silencing and $p < 10^{-7}$ for late silencing).

(D) As in **Figure 2D**, but showing percentage of change in raw reaction times instead of onset-corrected reaction times, in control (grey) and LM-silencing (purple) trials. P-values from two-sided Wilcoxon rank-sum test ($p = 0.036$ for early silencing and $p = 0.029$ for late silencing).

(E) As in **Figure 2E,** but showing percentage of change in the raw behavioral d-primes instead of the onset-corrected d-primes, in control (grey) and LM-silencing (purple) trials. P-values are from Wilcoxon two-sided signed-rank test (p = 0.622 for early silencing and p = 0.258 for late silencing).

(F) Average firing rate of V1 neurons (across trials and neurons), in the time windows corresponding to those used in **Figure 2F,** in control (grey) and LM-silencing (purple) trials**.** P-values are from Wilcoxon two-sided signed-rank test (p = 0.966 for early silencing and p = 0.397 for late silencing onsets). Error bars depict the standard error of the mean (s.e.m) in all panels.

Figure S6

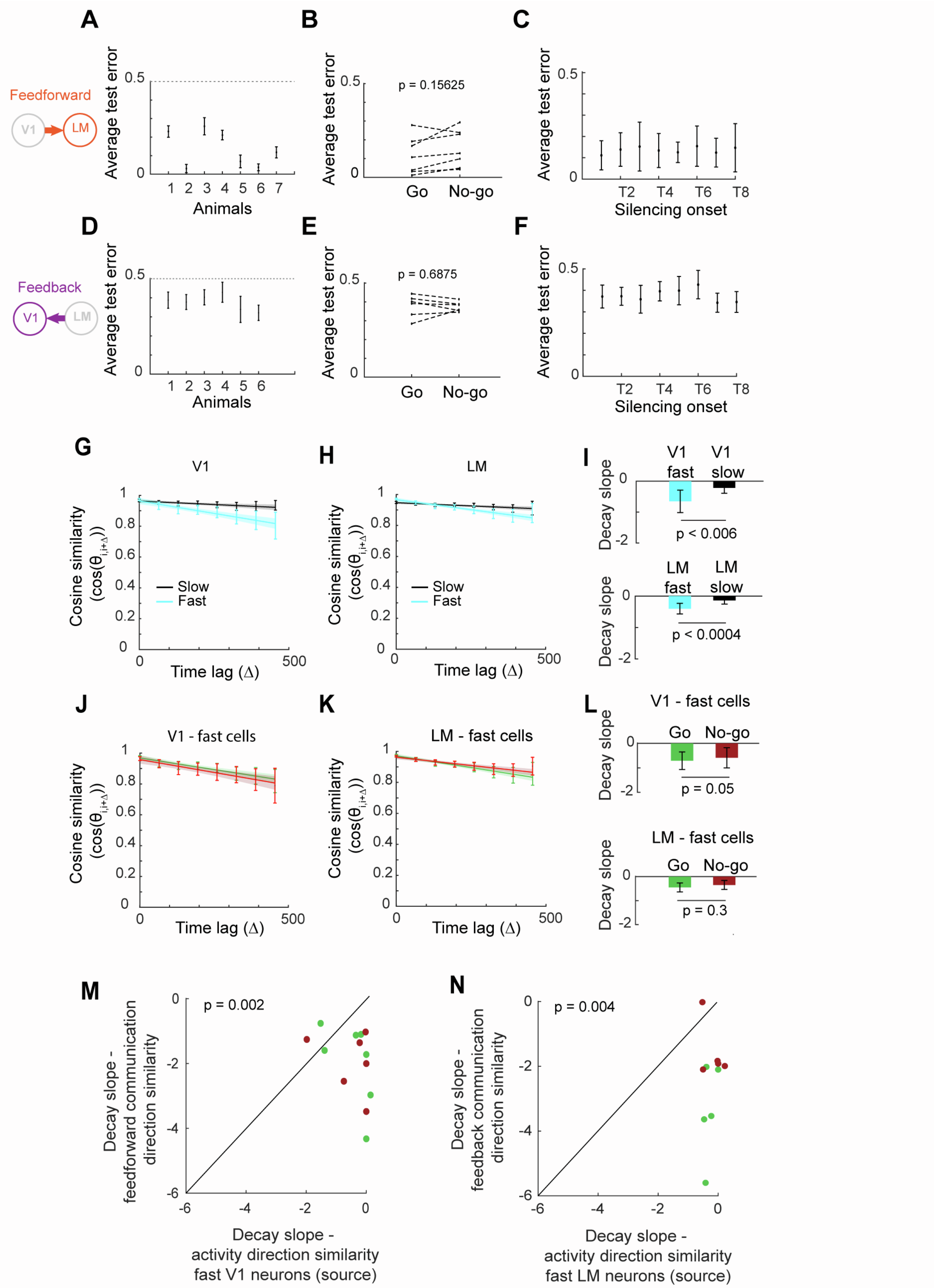

A
B
C
Feedforward
V1
LM
Average test error
0.5
0
1 2 3 4 5 6 7
Animals
p = 0.15625
Go
No-go
T2 T4 T6 T8
Silencing onset
D
E
F
Feedback
V1
LM
1 2 3 4 5 6
Animals
p = 0.6875
G
H
I
V1
LM
Cosine similarity
Slow
Fast
Time lag (Δ)
500
V1 fast
V1 slow
Decay slope
p < 0.006
LM fast
LM slow
p < 0.0004
J
K
L
V1 - fast cells
LM - fast cells
p = 0.05
p = 0.3
M
N
p = 0.002
Decay slope - feedforward communication direction similarity
Decay slope - activity direction similarity fast V1 neurons (source)
p = 0.004
Decay slope - feedback communication direction similarity
Decay slope - activity direction similarity fast LM neurons (source)

Figure S6. LDA and communication direction controls. Related to Figure 4 and STAR Methods.

(A) Cross-validated performance of the regularized linear discriminant analysis (LDA) for distinguishing LM population activity in control trials from that in V1 silencing trials. Y axis shows the percentage of misclassified trials (pooled across go and no-go stimuli) in the test data set using 10-fold cross validation for each individual animal.

(B) Cross-validated performance of the regularized linear discriminant analysis for distinguishing LM population activity in control trials from that in V1 silencing trials during go and no-go stimuli ($p = 0.156$, two-sided Wilcoxon signed-rank test). Points connected by dotted lines indicate individual animals.

(C) Cross-validated performance of the regularized linear discriminant analysis for distinguishing LM population activity in control trials from that in V1 silencing trials in the eight silencing time windows with varying onset times, averaged across animals ($n = 7$ animals). Performance was similar for the eight silencing time windows (one-way ANOVA $p = 0.99$).

(D) As in (A) but for distinguishing V1 population activity in control trials from that in LM silencing trials.

(E) As in (B) but for distinguishing V1 population activity in control trials from that in LM silencing trials.

(F) As in (C) but for distinguishing V1 population activity in control trials from that in LM silencing trials. ($n = 6$ animals, one-way ANOVA $p = 0.37$).

(G) Pair-wise cross-validated cosine similarity of activity directions in different time windows as a function of the time lag between them in two subpopulations of V1 neurons, the 20% of neurons with the most time-varying activity (based on the standard deviation over time in their z-scored activity, 'fast', blue), and the 20% of neurons with the least time-varying activity ('slow', black). Data was pooled across go and no-go stimuli ($n = 13$ mice).

(H) as in (G) but for LM cells.

(I) Initial slope (slope between lag 0 and lag 1) of the decay over time lags of fast-changing and slow-changing V1 subpopulations in (G, H). Error bars depict the 95% confidence interval of the mean (2×s.e.m).

(J) Pair-wise cross-validated cosine similarity of activity directions in different time windows as a function of the time lag between them in fast-changing V1 cells (20% of V1 neurons with the most time-varying activity) during go (green) and no-go trials (red).

(K) As in (J) but for LM cells.

(L) Top, initial slopes (slopes between lag 0 and lag 1) of the decay over time lags of fast-changing V1 neurons (20% of V1 neurons with the most time-varying activity) in go (green) and no-go (red) trials. Error bars depict the 95% confidence interval of the mean (2×s.e.m). Bottom, as on top, but for the fast-changing LM neurons.

(M) Relationship between the initial slopes (slopes between lag 0 and lag 1) of the decay over time lags of feedforward communication direction similarities and of activity direction similarities in fast-

changing V1 neurons (20% of V1 neurons with the most time-varying activity, see STAR Methods) in go (green) and no-go (red) trials for individual animals (n = 7 animals). In order to make the maximum dimensionality of communication directions comparable to those of the source area activity directions, the communication directions were re-calculated from a randomly selected subpopulation of 20% of target neurons (see STAR Methods).

(N) Relationship between the initial slopes (slopes between lag 0 and lag 1) of the decay over time lags of feedback communication direction similarities and of activity direction similarities in fast-changing LM neurons (20% of LM neurons with the most time-varying activity, see STAR Methods) in go (green) and no-go (red) trials for individual animals (n = 6 animals). In order to make the maximum dimensionality of communication directions comparable to those of the source area activity directions, the communication directions were re-calculated from a randomly selected subpopulation of 20% of target neurons (see STAR Methods).

Figure S7

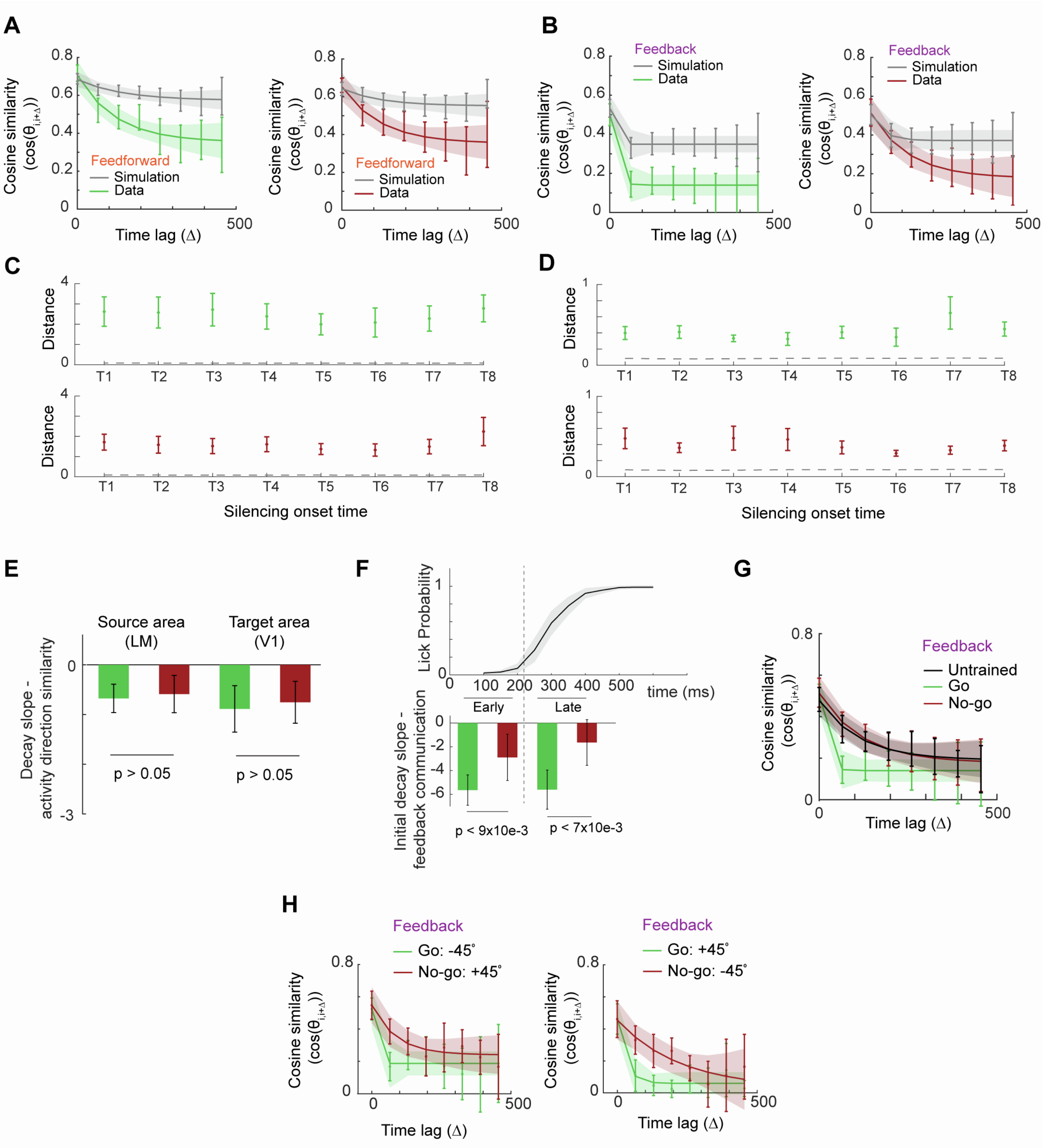

A
Cosine similarity (cos(θi,i+Δ))
Feedforward
Simulation
Data
Time lag (Δ)
B
Feedback
Simulation
Data
Time lag (Δ)
C
Distance
T1 T2 T3 T4 T5 T6 T7 T8
Silencing onset time
D
Distance
T1 T2 T3 T4 T5 T6 T7 T8
Silencing onset time
E
Decay slope - activity direction similarity
Source area (LM)
Target area (V1)
p > 0.05
p > 0.05
F
Lick Probability
time (ms)
Early
Late
Initial decay slope - feedback communication
p < 9x10e-3
p < 7x10e-3
G
Cosine similarity (cos(θi,i+Δ))
Feedback
Untrained
Go
No-go
Time lag (Δ)
H
Feedback
Go: -45°
No-go: +45°
Go: +45°
No-go: -45°
Time lag (Δ)

Figure S7. Modulation of population-level communication over time. Related to Figure 4.

(A) Cross-validated cosine similarity of pairs of feedforward communication directions (influence of V1 silencing on LM activity) in different time windows as a function of the time lag between them during the go (left, green) and no-go stimulus (right, red) and the corresponding cross-validated cosine similarity functions from the simulated dataset with time-invariant feedforward influences (grey). Error bars depict the 95% confidence interval of the mean (2×s.e.m). Lines depict exponential fits, shading shows 95% prediction bounds of the fits.

(B) As in (A) but for feedback communication directions (influence of LM silencing on V1 activity).

(C) Top, the magnitude of the feedforward influence of V1 on LM population activity in go trials (green) for the different silencing onset times. The magnitudes were calculated at each silencing time window as the Bhattacharyya distance between the LM population activity in control trials and V1 silencing trials (see STAR Methods). Error bars represent the standard error of the mean (s.e.m). The distance was similar across the eight silencing time windows (one-way ANOVA, $p = 0.99$). The grey dashed line represents the noise level (see STAR Methods). Bottom, as in top, but for no-go trials (red, one-way ANOVA, $p = 0.85$).

(D) As in (C), but for the magnitude of the feedback influence of LM on V1 population activity. Bhattacharyya distance in go trials shown in green (top, one-way ANOVA, $p = 0.45$), and in no-go trials shown in red (bottom, one-way ANOVA, $p = 0.80$).

(E) Initial slope (slope between lag 0 and lag 1) of the decay over time lags between activity directions during go (green) and no-go (red) trials, describing population activity in area LM (left) and V1 (right) in experiments in which LM silencing was performed ($n = 6$ animals). Error bars depict the 95% confidence interval of the mean (2×s.e.m).

(F) Top, cumulative lick probability in correct Go trials over time from stimulus onset (averaged over trials and mice). Bottom, initial decay slope of communication direction similarity (between time lags 0 and lag 1, similar to **Figure 4L**), for LM feedback influences on V1 activity, measured early during visual stimulus presentation (before most licks, silencing onsets 56, 123 and 189 ms after stimulus onset) and in a late period (during licking, 256, 323 and 390 ms after stimulus onset). Error bars depict the 95% confidence interval of the mean (2×s.e.m).

(G) Cross-validated cosine similarity of pairs of feedback communication directions in different time windows as a function of the time lag between them during grating stimuli presentation in untrained animals (black, passively viewing the visual stimuli), compared to trained animals during go (green) and no-go (red) stimuli (similar to **Figure 4F**. Error bars depict the 95% confidence interval of the mean (2×s.e.m). Lines depict exponential fits, shading shows 95% prediction bounds of the fits.

(H) Similarity of feedback communication directions over time, as in **Figure 4F**, shown separately for mice trained on the -45°orientated grating as go stimulus (left, $n = 4$, exponential decay time constant, go trials: 3 ms, 0 to 22 ms 95% confidence interval, CI; no-go trials: 87 ms, 46 to 122 ms CI; $p = 0.06$, permutation test), and for mice trained on the +45° orientated grating as the go stimulus (right,

n = 2, exponential decay time constant, go trials: 29 ms, 1 to 60 ms 95% confidence interval, CI; no-go trials: 225 ms, 167 to 317 ms CI; $p < 10^{-6}$, permutation test).

Figure S8

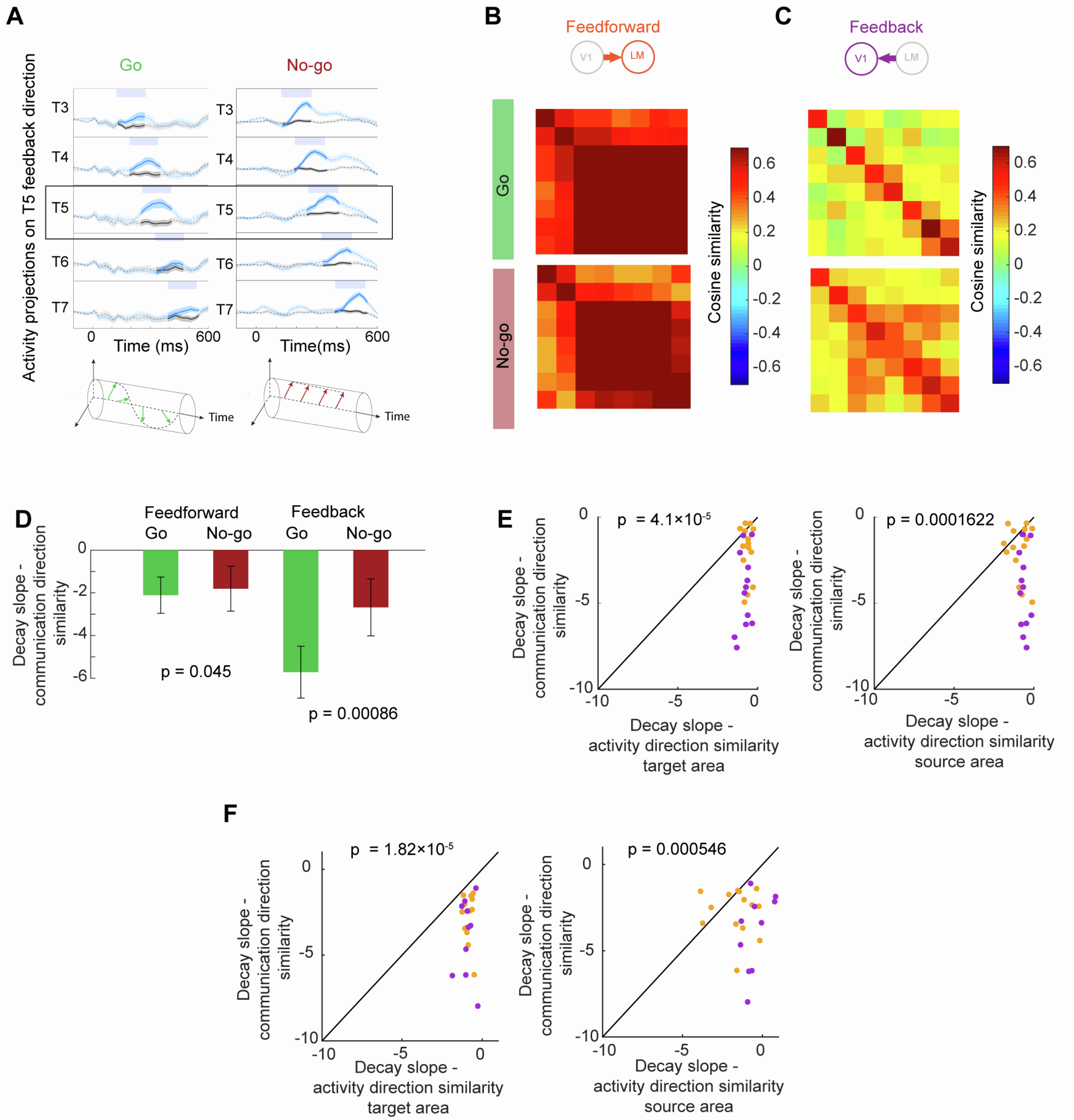
A
B
C
D
E
F
Go
No-go
Activity projections on T5 feedback direction
T3
T4
T5
T6
T7
0
600
Time (ms)
Time(ms)
Time
Feedforward
Feedback
V1
LM
Cosine similarity
0.6
0.4
0.2
0
-0.2
-0.4
-0.6
Decay slope - communication direction similarity
Feedforward
Feedback
p = 0.045
p = 0.00086
p = 4.1×10-5
p = 0.0001622
p = 1.82×10-5
p = 0.000546
Decay slope - activity direction similarity target area
Decay slope - activity direction similarity source area
-10
-5

Figure S8. Communication direction and activity direction controls. Related to Figure 4.

(A) Top, projections of V1 activity during the visual stimulus presentation in control (black) and LM silencing (blue) trials onto the feedback communication direction calculated for the silencing window at time T5 (256 ms). V1 activity in different silencing windows (T3, T4 shown above, T6 and T7 below) is then projected onto the same feedback communication direction calculated for T5 during go (left) and no-go trials (right). In no-go trials, the feedback direction at time T5 consistently separates control from silencing trials, not only at T5, but also at earlier and later silencing onset times. During go trials this separation between control and silencing trials decreases rapidly with increasing time intervals. This indicates more rapid changes in feedback directions over time in go, compared to no-go trials. Bottom, schematic illustrating the faster temporal re-orientation of the feedback communication direction in go (green) as opposed to no-go (red) trials.

(B) Cross-validated cosine similarity matrices for the feedforward communication directions (influence of V1 silencing on LM population activity) calculated using an alternative method. Instead of LDA, the difference between the means of population activity in control and silencing trials (without normalizing to the covariance matrix, see STAR Methods) was used to calculate communication directions.

(C) As in (B) but for feedback communication directions (influence of LM silencing on V1 population activity).

(D) Initial decay slope of communication directions in go (green) and no-go (red) trials, calculated from the difference between the means of population activity in control and silencing trials (corresponding to (B) and (C)) instead of using LDA. Error bars depict the 95% confidence interval of the mean (2×s.e.m). P-values from Wilcoxon two-sided signed-rank test for comparisons between go and no-go.

(E) Relationship between the initial slopes (slopes between lag 0 and lag 1) of the decay over time lags of communication direction similarities and of activity direction similarities in the target area (left), and in the source area (right), calculated from the difference between the means of population activity in control and silencing trials (corresponding to (B) and (C)) instead of using LDA. Orange and purple dots show data from individual animals during V1 silencing (feedforward influences) and LM silencing (feedback influences) experiments, respectively. P-values from two-sided Wilcoxon signed-rank tests.

(F) Relationship between the initial slopes (slopes between lag 0 and lag 1) of the decay over time lags of communication direction similarities and of activity direction similarities in the target area (left), and in the source area (right), calculated using baseline-subtracted activity (see STAR Methods). Orange and purple dots show data from individual animals during V1 silencing (feedforward influences) and LM silencing (feedback influences) experiments, respectively. P-values from two-sided Wilcoxon signed-rank tests.

Figure S9

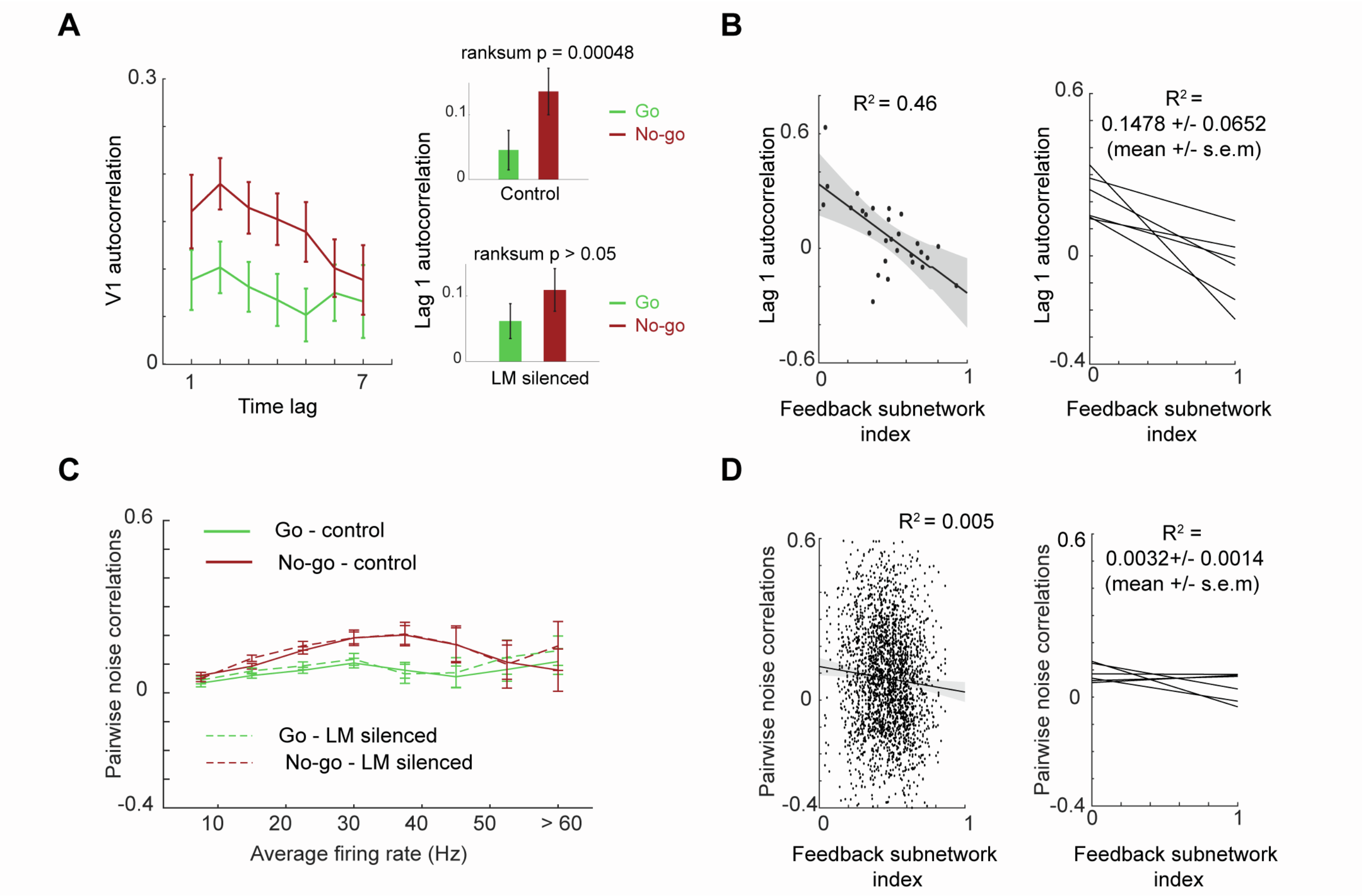

Figure S9. Pairwise noise correlations and autocorrelations of V1 cells. Related to Figure 5

(A) Left, Autocorrelation of V1 cells as a function of time lags, calculated for each cell separately and averaged across cells during go (green) and no-go (red) trials. Right, Autocorrelation of V1 neurons at time lag 1 (~65ms time lag) during go (green) and no-go (red) stimulus, in control trials (top) and during LM silencing (bottom). Error bars depict the 95% confidence interval of the mean (2×s.e.m). A comparison of autocorrelation strength in control and silencing trials is only possible at time lag 1, since silencing was not performed continuously but during short time windows.

(B) Left, Relationship between autocorrelation at time lag 1 (~65ms time lag) and the feedback influence index of individual V1 neurons of one example mouse in go trials. The feedback influence index quantifies how much a V1 neuron was influenced by feedback, calculated as its maximum coefficient in the feedback communication direction across the eight silencing time windows. Line represents the linear fit, shading depicts 95% prediction bounds of the fit. Right, linear fits as on the left for all mice. Each line represents a linear fit to the relationship between autocorrelation and feedback influence index of V1 neurons from one animal (n = 6).

(C) Average strength of noise correlations between pairs of V1 cells as a function of their average firing rates during go (green) and no-go (red) stimuli, when LM was silenced (dashed lines) and in the corresponding time windows in control trials (solid lines). Error bars depict the 95% confidence interval of the mean (2×s.e.m).

(D) Left, relationship between pairwise noise correlation of pairs of V1 cells and the average feedback influence index of the pair of one example mouse in go trials. Feedback influence index for each V1 neuron was calculated as in (B). Right, linear fits as in (C) for all mice (n = 6).
